# Supplementary material for: NXT2 is a key component of the RNA nuclear export factor complex in the human testis and essential for spermatogenesis
Source: Nat Commun. 2025 Jul 7;16:6254. doi: 10.1038/s41467-025-61463-0 (PMC12234887; doi:10.1038/s41467-025-61463-0)

Supplementary Information

Table of Contents

Supplementary Table 1. Antibody information.

Supplementary Table 2: Genetic variants identified in *NXT2* and *NXF3*.

Supplementary Table 3. Clinical data of infertile men with *NXT2*/*NXF3* variants.

Supplementary Table 4. Analysis of M3065's familial exome sequencing data does not identify other high impact genetic variants that co-segregate with the phenotype.

Supplementary Table 5. Primer information.

Supplementary Figure 1. NXT2/NXF3/NXT1 antibody validation.

Supplementary Figure 2. Pulldown of NXT2, NXF3 and NXT1 from human adult testis lysate

Supplementary Figure 3. Gene ontology (GO) analysis on at least 5-fold enriched proteins in the NXT2 pulldown.of human nuclear export factor genes derived from the Human Protein Atlas.

Supplementary Figure 4. Modular architecture of NXT1, NXT2, NXF1, NXF2 and NXF3.

Supplementary Figure 5. Western blot analysis of NXF2 and NXF3 proteins expressed from deletion constructs lacking the NTF2-like domain or RRM domain.

Supplementary Figure 6. The NXT2 protein region downstream of the NTF2-like domain is not essential for binding to NXF2 and NXF3.

Supplementary Figure 7. NXT1 binds to NXF2 and NXF3 *in vitro*.

Supplementary Figure 8. No NXT1 specific staining is detectable in human testicular tissue.

Supplementary Figure 9. The *NXT2* missense variant c.268G>T p.(Ala90Ser) in M2004 affects splicing at NXT2 exon 4 but does not impact protein expression.

Supplementary Figure 10. Differential interference contrast (DIC) microscopy of sperm from M2799 with LoF variant in *NXF3*.

Supplementary Figure 11. scRNA-seq shows expression of genes of the nuclear export factor family in fetal male germ cells and adult testis.

Supplementary Figure 12. Schematic overview of germ cell development with temporal expression of the *NXT2* gene and the testicular *NXF* genes depicted.

Supplementary References

Source data of Supplementary Figures

Supplementary Table 1. Antibody information.

**Antibody**	**Company, catalogue #, source species, clonality, LOT**	**Dilution, application, special protocol requirements**
**Primary Antibodies**		
α- α/β-Tubulin	Cell Signaling Technology, #2148, rabbit, polyclonal, 8	1:250 (ICC)
α-FLAG	Merck, #F3165, mouse, monoclonal, SLCJ3741	1:1500 (Western blot)
α-GAPDH	Cell Signaling Technology, #5174, rabbit, monoclonal, 8	1:1500 (Western blot)
α-HA	Roche, #11867423, rat, monoclonal, 65506600	1:2000 (Western blot)
α-IgG	Cell Signaling Technology, #2729, rabbit, polyclonal, I5006	5 µg (pulldown of testicular lysates)
α-MAGEA4	Abcam, #ab139297, mouse, monoclonal, GR3177245-18	1:500 in 5 % BSA/TBS (IHC)
α-NXF3	Merck, #HPA046757, rabbit, polyclonal, R43903	1:500 (Western blot)<br>1:250 (ICC)
α-NXT2	Merck, #HPA072010, rabbit, polyclonal, R102934	5 µg (pulldown of testicular lysates)<br>1:1000 (Western blot)<br>1:50 in 5 % BSA/TBS (IHC)
α-NXT1	Proteintech, #67680, mouse, monoclonal, 10017134	10 µg (pulldown of testicular lysates)<br>1:2000-1:5000 (Western blot)<br>1:200 in 25% goat serum in 5 % BSA/TBS (IHC)
α-SMA	Sigma-Aldrich, # A2547, mouse, monoclonal, 0000106190	1:1000 in 5% BSA/TBS
α-DDX4	Abcam, #ab13840, rabbit, polyclonal, GR3274948-1	1:2000 in 5% BSA/TBS
α-SOX9	Sigma Aldrich, #AB5535, rabbit, polyclonal, 3481414	1:1000 in 5 % BSA/TBS (IHC)
**Secondary Antibodies**		
α-mouse	Abcam, #5886, goat, monoclonal, GR195138-16	1:100 in 5 % BSA/TBS (IHC)

α-mouse IgGκ BP-HRP	Santa Cruz Biotechnology, #sc-516102, donkey, monoclonal, F1620	1:1000 (Western blot)
α-rabbit	Abcam, #ab6012, goat, monoclonal, GR3353084-9	1:100 in 5 % BSA/TBS (IHC)
α-rabbit Alexa Fluor Plus 488	Thermo Scientific, #A32790, donkey, monoclonal, VE306220	1:250 (ICC)
α-rabbit IgG HRP	Santa Cruz Biotechnology, #sc-2357, mouse, monoclonal, E0819	1:1000 (Western blot)
α-rat HRP	Merck, #A9037, goat, monoclonal	1:2000 (Western blot)

Supplementary Table 2: Genetic variants identified in *NXT2* and *NXF3*.

<table>
<tr><th>Subject ID</th><th>Gene</th><th>Variant g.*</th><th>Variant c.</th><th>Variant p.</th><th>Genotype</th><th>MAF (gnomAD v2.1.1/ v4.1.0)</th><th>LoF o/e fraction (gnomAD v2.1.1)</th><th>LoF o/e upper bound fraction (LOEUF) (gnomAD v2.1.1)</th></tr>
<tr><td>M2004</td><td>NXT2</td><td>chrX:108,784,704G>T</td><td>c.268G>T</td><td>p.(Ala90Ser)</td><td>hem.</td><td>0.00002909 /0.0001080</td><td rowspan="3">0</td><td rowspan="3">0.51</td></tr>
<tr><td>M3065</td><td>NXT2</td><td>chrX:108,784,790dup</td><td>c.354dup</td><td>p.(Asp119*)</td><td>hem.</td><td>-</td></tr>
<tr><td>RU00584</td><td>NXT2</td><td>chrX:108,765,361-108,807,493del</td><td colspan="2"></td><td>hem.</td><td>-</td></tr>
<tr><td>M2799</td><td>NXF3</td><td>chrX:102,337,247G>T</td><td>c.826G>T</td><td>p.(Gly276*)</td><td>hem.</td><td>-</td><td>0.33</td><td>0.6</td></tr>
</table>

* Chromosomal positions are referring to Human GRCh37/hg19

Supplementary Table 3: Clinical data of infertile men with *NXT2*/*NXF3* variants.

Individual	Genotype	Andrological phenotype	Testicular phenotype (PAS/MAGEA4),<br>TESE outcome
M2004	*NXT2*:<br>g.108,784,704<br>c.268G>T<br>p.(Ala90Ser)	FSH: 9.4<br>LH: 3.2<br>T: 15.4<br>TV: 20/20<br>Azoospermia	98 % SCO, 2 % TS/<br>no germ cells detected<br>no sperm retrieved
RU00584	*NXT2*:<br>g.108,765,361-108,807,493del	FSH: 14.9<br>LH: 4.7<br>T: 9.6<br>TV: 10/12<br>Azoospermia	SCO/no germ cells detected<br>no sperm retrieved; some spermatozoa seen during inspection of biopsy-derived cell suspension - due to their very abnormal morphology material was not cryopreserved
M3065	*NXT2*:<br>g.108,784,790dup<br>c.354dup<br>p.(Asp119*)	FSH: 63.8<br>LH: 9.7<br>T: 12.7<br>TV: 3/3<br>Azoospermia	70 % SCO, 30 % TS/<br>focal spermatogenesis in ~7 % (11/161) of seminiferous tubules<br>no sperm retrieved
M2799	*NXF3*:<br>g.102,337,247<br>c.826G>T<br>p.(Gly276*)	FSH: 4.7<br>LH: 5.8<br>T: 20.5<br>TV: 17/18<br>Oligoasthenoteratozoospermia<br>(>85 % immotile sperm; 0% normal sperm morphology)	no biopsy performed

TESE: testicular sperm extraction, FSH: follicle stimulating hormone (IU/L), LH: luteinizing hormone (IU/L), T: testosterone (nmol/L), TV: testicular volume right/left (mL), SCO: Sertoli cell-only, TS: tubular shadows | Reference values: FSH 1-7 IU/L, LH 2-10 IU/L, T >12 nmol/L, TV >12 mL per testis.

Supplementary Table 4. Analysis of M3065's familial exome sequencing data does not identify other variants that co-segregate and are candidates to cause the phenotype.

Gene	Chr.	Variant c.	Variant p.	Consequence	MAF (gnomAD)	CADD
ELF4	X	c.1810C>T	p.(Arg604Cys)	missense variant	0.26 %	20.7
NLGN3	X	c.63C>A	p.(Ser21Arg)	missense variant	0.01 %	17.35
RIPPLY1	X	c.362A>G	p.(Asn121Ser)	missense variant	0.08 %	19.26
TIMP1	X	c.195G>T	p.(Met65Ile)	missense variant	0.03 %	18.83

Supplementary Table 5: Primer information.

**Sanger Sequencing**	
NXT2 c.268G>T	
Forward	5’-CCAGTGGAGATGGAGCAAGG-3’
Reverse	5’-ATCCAGCCCTGAAACAGCAT-3’
NXT2 c.354dup	
Forward	5'-CCAGTGGAGATGGAGCAAGG-3'
Reverse	5'-GTTTCTCGCTGCTTTTGGTGT-3'
NXF3 c.826G>T	
Forward	5'-TGCAGAAGGGCGCTATCAAA-3'
Reverse	5'-AGAAAAAGGGGGAGGAGGGT-3'
**Minigene assay**	
NXT2 c.268G>T (Initial amplification)	
Forward	5’-CACCACACTAAAACTGGCTTGGGG-3'
Reverse	5'-GACATTTCAGGGTCCAGCAG-3'
Minigene exon primers (Rat *INS2* exons 3 and 4)	
Forward	5'-CCTGCTCATCCTCTGGGAGC-3'
Reverse	5'-AGGTCTGAAGGTCACGGGCC-3'
**Cloning of cDNA constructs**	
NXT2 (NM_018698.5)	
Forward	5’-GGTGGTAAGCTTATGAGAAAATACAGAAGCCACTGGTC-3’
Reverse	5’-GGTGGTCTCGAGTTAACTACTAGACCAATCTTGAAAACGG-3’
NXF2 (NM_022053.4)	
Forward	5’-GGTGGTAAGCTTATGTGCTCTACTCTAAAGAAGTGTGGG-3’
Reverse	5’-GGTGGTCTCGAGTTAGGAGATTTGCTTGAAGGCCTCCGCG-3’
NXF3 (NM_022052.2)	
Forward	5’-GGTGGTGAATTCATGTCACTGCCTTCAGGACACACTACG -3’
Reverse	5’-GGTGGTCTCGAGTTACGAAGGCAGCATTTTCTGCTGCTCC-3’
NXT1 (NM_013248.3)	
Forward	5’-GGTGGTAAGCTTGCGTCTGCGTGGAGACCGGCTGGCCG-3’
Reverse	5’-GGTGGTCTCGAGCTAGCTGGCCCAGTCCTGGAAGCGGAAG-3’
**Primers for cloning of 5’ Kozac sequence (phosphorylated)**	
NXF2	
Forward	5’-ATGTGCTCTACTCTAAAGAAGTG-3’
Reverse	5’-GGTGGCAAGCTTAAGTTTAAACGCTAGCCA-3’
NXF3	
Forward	5’-ATGGACTACAAAGACGATGACGA-3’
Reverse	5’-GGTGGCGAATTCCACCACACTGGACTAGTG-3’
**Primers for cloning of tags and for introducing deletions (phosphorylated)**	
NXT2 (HA tag)	
Forward	5’-AGAAAATACAGAAGCCACTGGTC-3’
Reverse	5’-AGCGTAATCTGGAACATCGTATGGGTACATAAGCTTAAGTTTAA-ACGCTAG-3’

NXF2 (3xFLAG tag)	
Forward	5’-CACGACATCGACTACAAGGACGACGACGACAAGTGATAAGTCTA-GAGGGCCCGTTTAAACC-3’
Forward 2	5’-GACTACAAGGACCACGACGGTGACTACAAGGACCACGACATCG-ACTACAAGGACGACG-3’
Reverse	5’-GGAGATTTGCTTGAAGGCCTCC-3’
NXF3 (FLAG tag)	
Forward	5’-TCACTGCCTTCAGGACACACTAC-3’
Reverse	5’-CTTGTCGTCATCGTCTTTGTAGTCCATGAATTCCACCACACTGG-ACTA-3’
NXT1 (HA tag)	
Forward	5’-GCATCTGTGGATTTCAAGACCTA-3’
Reverse	5’AGCGTAATCTGGAACATCGTATGGGTACATCTCTGGTTCTATGGAA GGAGG-3’
NXT2 (AA137-142del)	
Forward	5’-AAAACGGAAGCAATCACTTGCAA-3’
Reverse	5’-TAACTCGAGTCTAGAGGGCCCGT-3’
NXF2 (NTF2LD_del)	
Forward	5’-AGGGATGCCAGCCCCCAAGAGAC-3’
Reverse	5’-ATGCTTTAGGGTCTCAGATCCAG-3’
NXF3 (NTF2LD_del)	
Forward	5’-CGGGATACCAGCCACCAAGGGAC-3’
Reverse	5’-ATTCTTCAACATCTCAGATCCAA-3’
NXF2 (RRMD_del)	
Forward	5’-TCTGTGAAGAATAAGTTGAAGCC-3’
Reverse	5’-GTTCCTTGTGTATCCATCCTGTG-3’
NXF3 (RRMD_del)	
Forward	5’-TTTGTGCACAGGGAGCTGAAGTC-3’
Reverse	5’-GCTCCCTAAGGTCCCATCCGGCA-3’
**Primers for site-directed-mutagenesis**	
NXT2 (c.268G>T)	
Forward	5’-CGGTCACTAACCAGGCTGTATCTGGAC-3’
Reverse	5’-GGTTAGTGACCGTCTTCTTTTATCCATTGTCTC-3’
NXT2 (c.354dup)	
Forward	5’-CTAAATAATTTTTTTTGACACATTGCCTTCTAGTGAGTTC-3’
Reverse	5’-GTGTCAAAAAAAATTATTTAGGGCATCCAGCCCTG-3’
NXF3 (c.826G>T)	
Forward	5’-AGCCATGAGAGAAGTGTGCGGACAGAAGCC-3’
Reverse	5’-ACTTCTCTCATGGCTCTATCCCTTTCCACTTGTC-3’

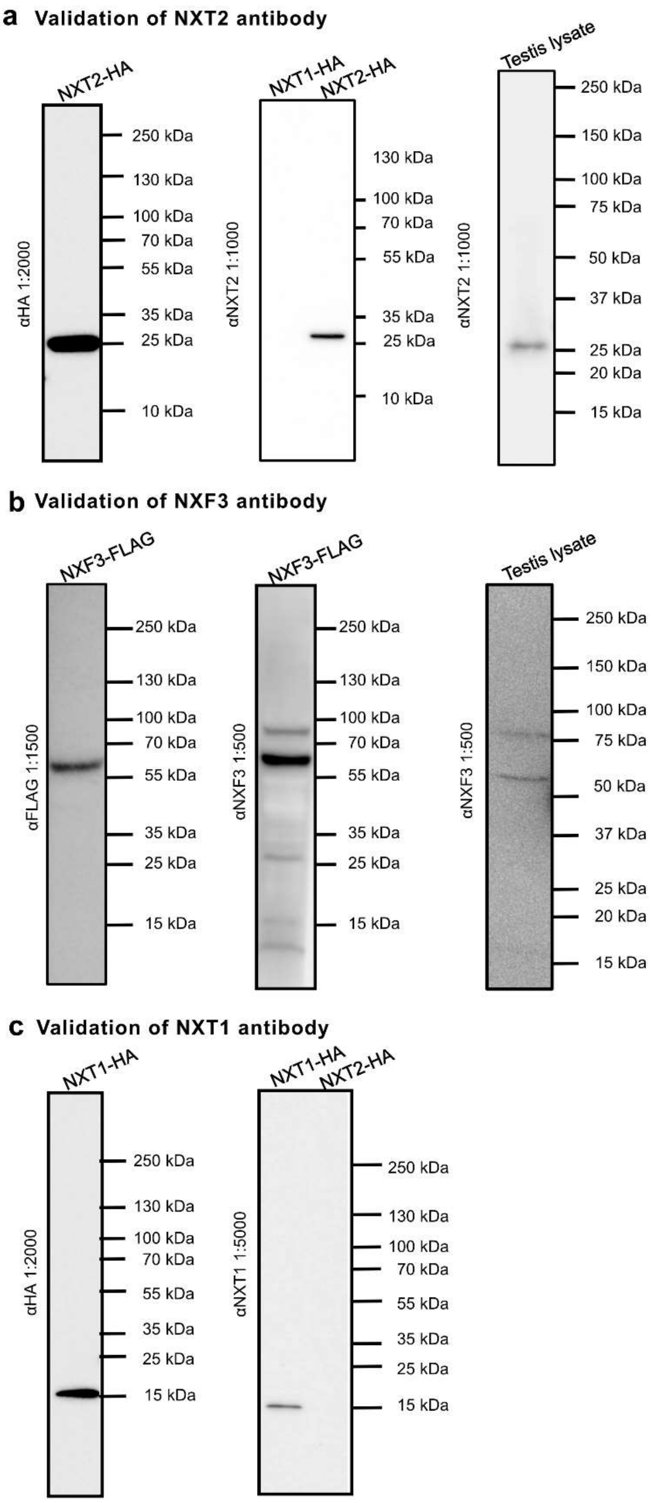

Supplementary Figure 1. NXT2/NXF3 antibody validation. a. Western blot analysis of protein lysates isolated from HEK cells transfected with HA-tagged NXT2 indicating specific staining of the recombinant NXT2 protein with both, the HA-tag antibody and a polyclonal NXT2 antibody (HPA072010). Both approaches resulted in a distinct band correlating with the expected size of NXT2 (~26 kDa). HA-tagged NXT1 is not detected by the NXT2 antibody. In Western blot analysis of lysates from native human testicular tissue a specific band of 26 kDa was detected with the NXT2 antibody (HPA072010). b. Western blot analysis of protein lysates

isolated from HEK cells transfected with FLAG-tagged NXF3 indicating specific staining of the recombinant NXF3 protein with FLAG-tag antibody and a polyclonal NXF3 antibody (HPA046757). Both Western blots resulted in a distinct band with the expected size of ~60 kD. In native testicular tissue lysates, NXF3 can be detected using the NXF3 specific antibody (HPA046757). c. Western blot analysis of protein lysates isolated from HEK cells transfected with HA-tagged NXT1 indicating specific staining of the recombinant NXT1 protein with HA-tag antibody and a monoclonal NXT1 antibody (Proteintech, 67680). Both Western blots resulted in a distinct band with the expected size of ~16 kD. All Western blots were repeated at least twice (N=2). Source data are provided as a Source Data file.

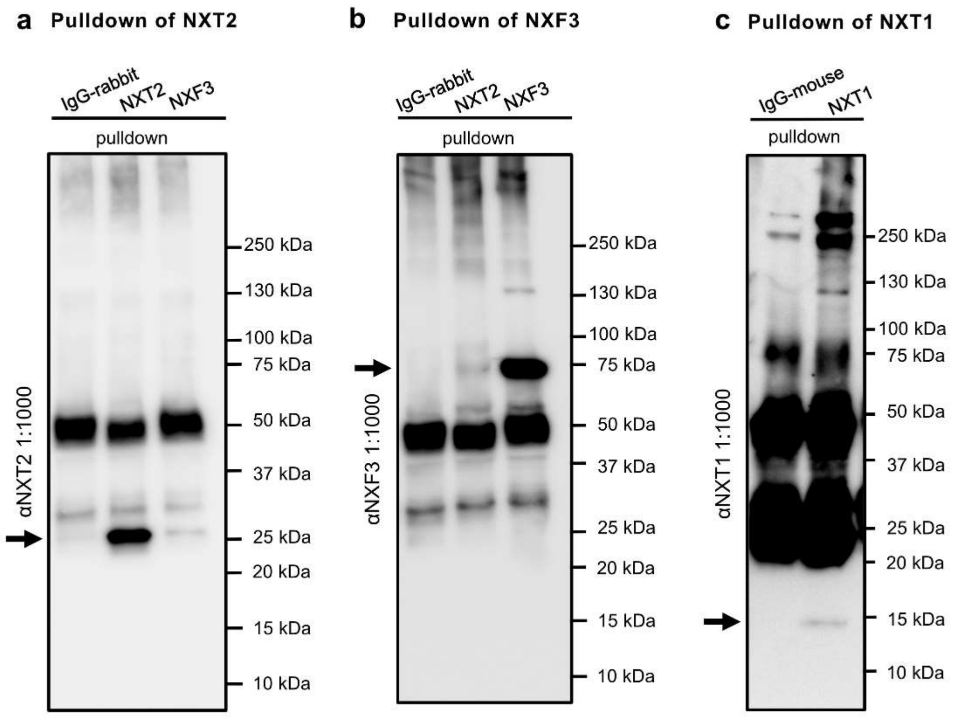

Supplementary Figure 2. Pulldown of NXT2, NXF3 and NXT1 from human adult testis lysate. a. Western blot from a pulldown of NXT2 from testicular lysate derived from a transgender donor with full spermatogenesis (lysate 1). NXT2 (arrow) is specifically detected in the NXT2 pulldown sample but not in the IgG isotype control. b. Western blot from a pulldown of NXF3 (from lysate 1). NXF3 (arrow) is specifically detected in the NXF3 pulldown sample and not in the IgG isotype control. c. Western blot from a pulldown of NXT1 (from lysate 1). Only when the blot is overexposed a faint NXT1-specific signal (arrow) is detected in the NXT1 pulldown. In all samples bands at 50 and 30 kDa refer to the IgG heavy and light chain, respectively. All Western blots were repeated three times (N=3). Source data are provided as a Source Data file.

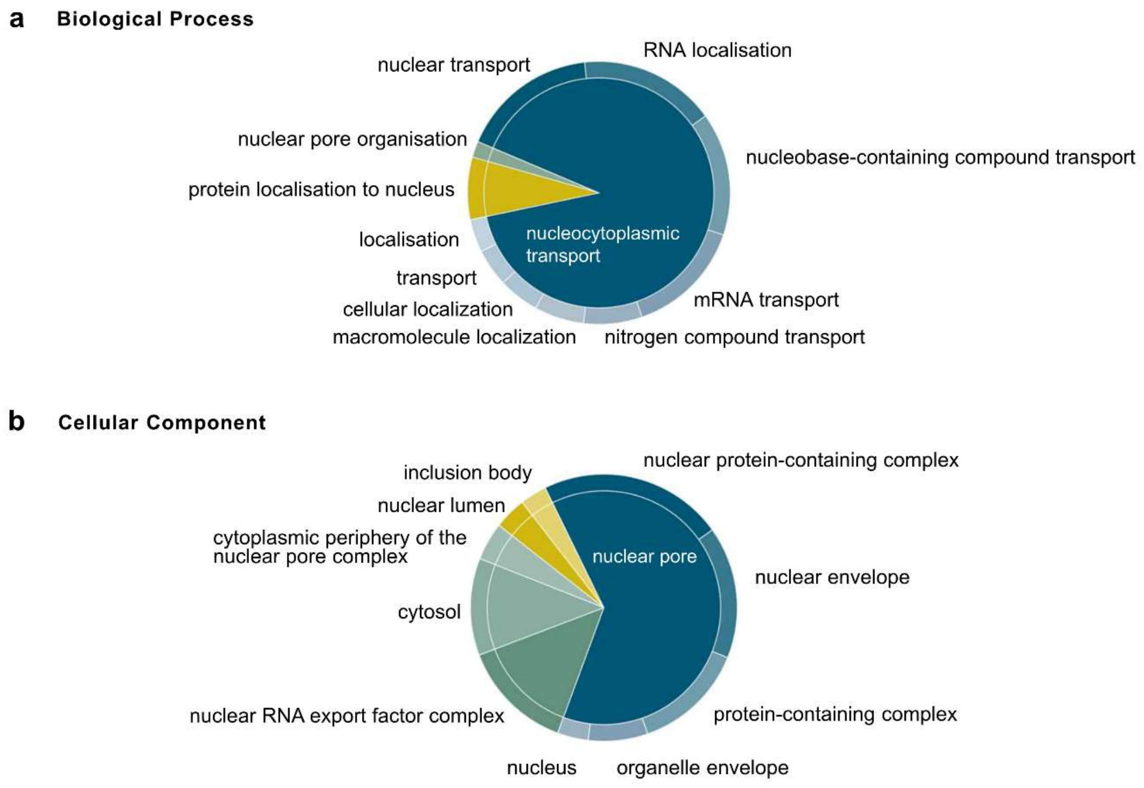

Supplementary Figure 3. Gene ontology (GO) analysis on at least 5-fold enriched proteins in the NXT2 pulldown. a. Gene Ontology (GO) term analysis of significantly enriched proteins highlights biological processes associated with NXT2 and its binding partners in a two-tiered-hierarchy. Top GO term was “nucleocytoplasmic transport”. Terms which are hierarchically under and belong to “nucleocytoplasmic transport” are listed in the outer circular segment. b. Name and relative proportion of GO terms describing the cellular components associated with the NXT2 interactome. Top cellular component is the “nuclear pore”. GO terms hierarchically under “nuclear pore” are indicated in the outer ring.

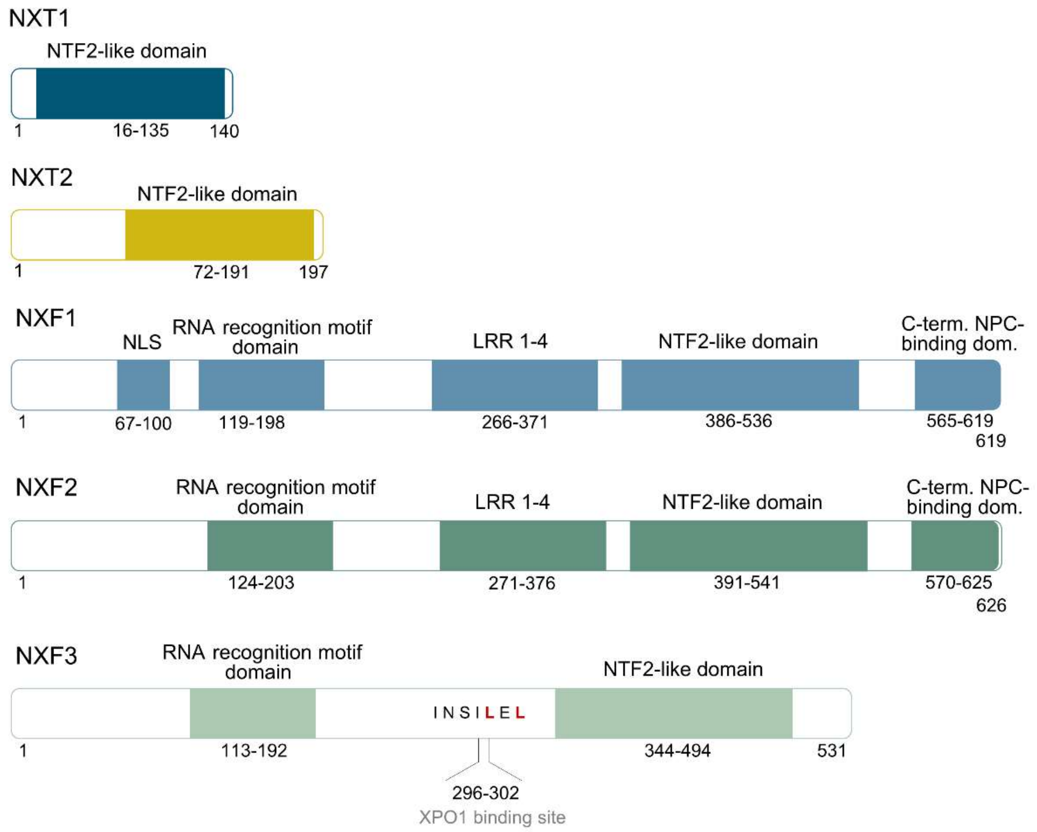

Supplementary Figure 4. Modular architecture of NXT1, NXT2, NXF1, NXF2, and NXF3. NXT1 and NXT2 consist almost exclusively of a nuclear transport factor 2-like domain (NTF2LD). NXF1 exhibits a nuclear localization signal (NLS), an RNA recognition motif domain (RRMD), four leucine-rich repeats (LRR), an NTF2-like domain and a C-terminal nuclear pore binding domain (C-term. NPC-binding domain). NXF2 shows a similar protein structure. NXF3 is lacking the C-terminal NPC-binding domain but gained an exportin (XPO1) binding site.

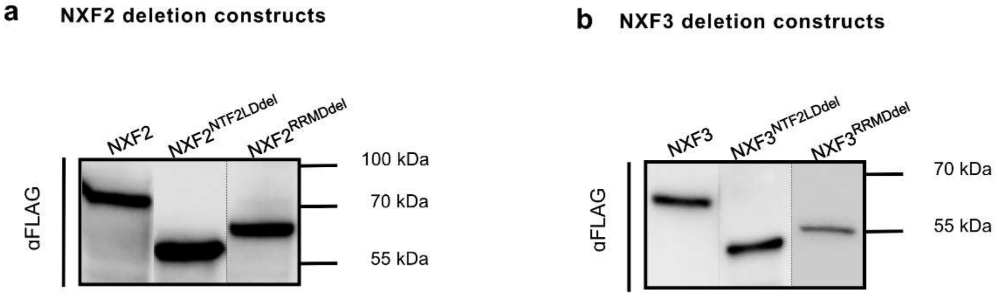

Supplementary Figure 5. Western blot analysis of NXF2 and NXF3 proteins expressed from deletion constructs lacking the NTF2-like domain or RRM domain. a. The deletion of the NTF2-like domain and the RRM domain from the NXF2-FLAG expression construct results in the expression of truncated NXF2 proteins, when overexpressed in HEK cells, as visualized using a FLAG antibody in a Western blot. Calculated molecular weights are ~72 kDa for full-length NXF2-FLAG, ~55 kDa for $NXF2^{NTF2LDdel}$-FLAG, and ~63 kDa for $NXF2^{RRMDdel}$-FLAG. b. Western blot analysis of lysates derived from overexpression of NXF3 deletion constructs in HEK cells demonstrating expression of truncated FLAG-tagged NXF3 proteins compared to the wildtype. Calculated molecular weights are ~60 kDa for full-length NXF3-FLAG, ~43 kDa for $NXF3^{NTF2LDdel}$-FLAG, and ~51 kDa for $NXF3^{RRMDdel}$-FLAG (N=3). Source data are provided as a Source Data file.

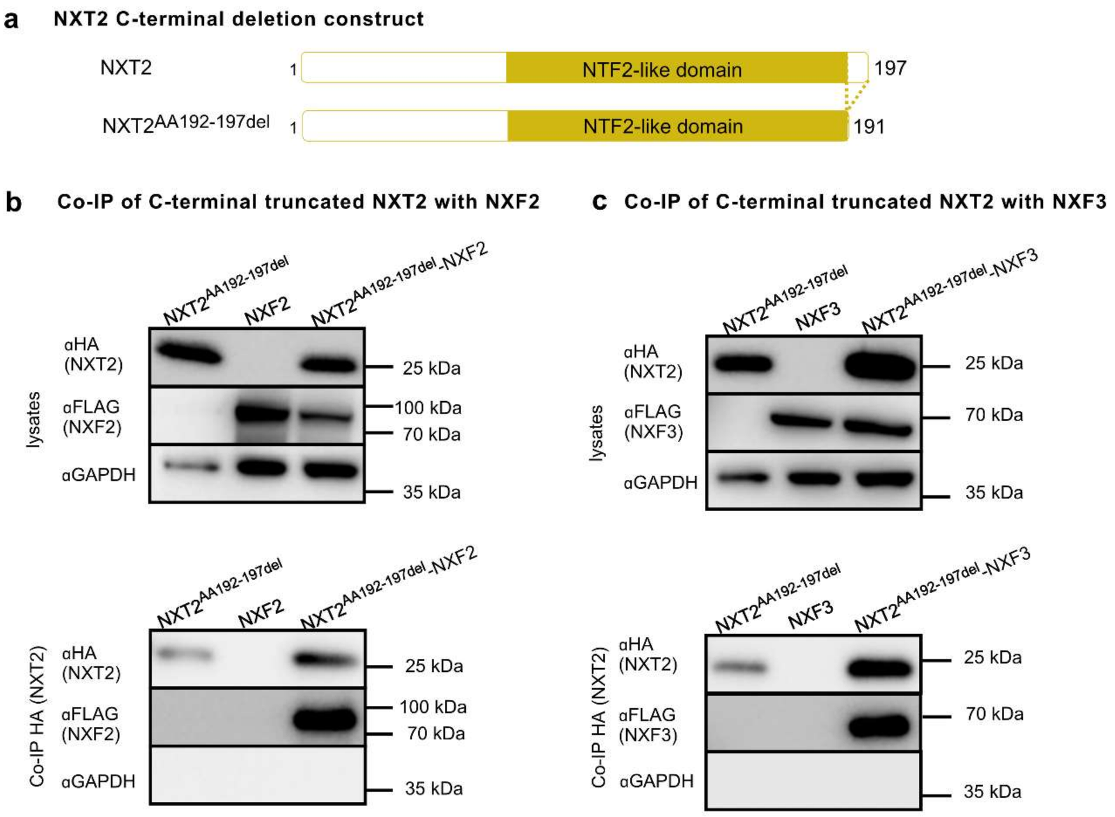

Supplementary Figure 6. The NXT2 protein region downstream of the NTF2-like domain is not essential for binding to NXF2 and NXF3. a. Deletion of six C-terminal amino acids of NXT2-HA, located downstream of the predicted NTF2-like domain (amino acids 192-197 in NM_018698.5) truncates the protein. b. The C-terminal truncated NXT2-HA protein is still able to bind to NXF2-FLAG as demonstrated by Co-IP of protein lysates obtained from NXT2 and NXF2 constructs overexpressed in HEK293T cells and visualized in a Western blot. The top Western blot refers to protein lysates, while the bottom blot is derived from Co-IP. c. The interaction between NXT2$^{AA192-197del}$-HA and NXF3-FLAG persists even without the C-terminus of NXT2. Lysates are visualized at the top, Co-IP-samples at the bottom. Western blots demonstrate representative results of at least three replicates (N=3). Source data are provided as a Source Data file.

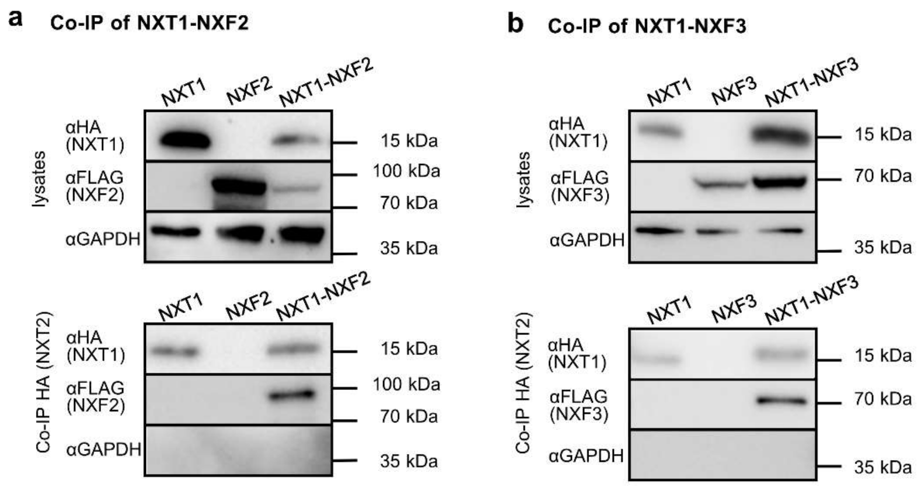

Supplementary Figure 7. NXT1 binds to NXF2 and NXF3 *in vitro*. a. Co-IP of protein lysates obtained from HA-tagged NXT1 and FLAG-tagged NXF2 constructs overexpressed in HEK293T cells and analyzed in a Western blot. The top Western blot refers to input protein lysates, while the bottom blot is derived from the Co-IP. NXF2-is only present in Co-IP if NXT1 is co-expressed. b. Co-IP of protein lysates obtained from HA-tagged NXT1 and FLAG-tagged NXF3 constructs overexpressed in HEK293T cells and analyzed in a Western blot. The top Western blot refers to input protein lysates, while the bottom blot is derived from the Co-IP. Western blots demonstrate representative results of at least three replicates (N=3). Source data are provided as a Source Data file.

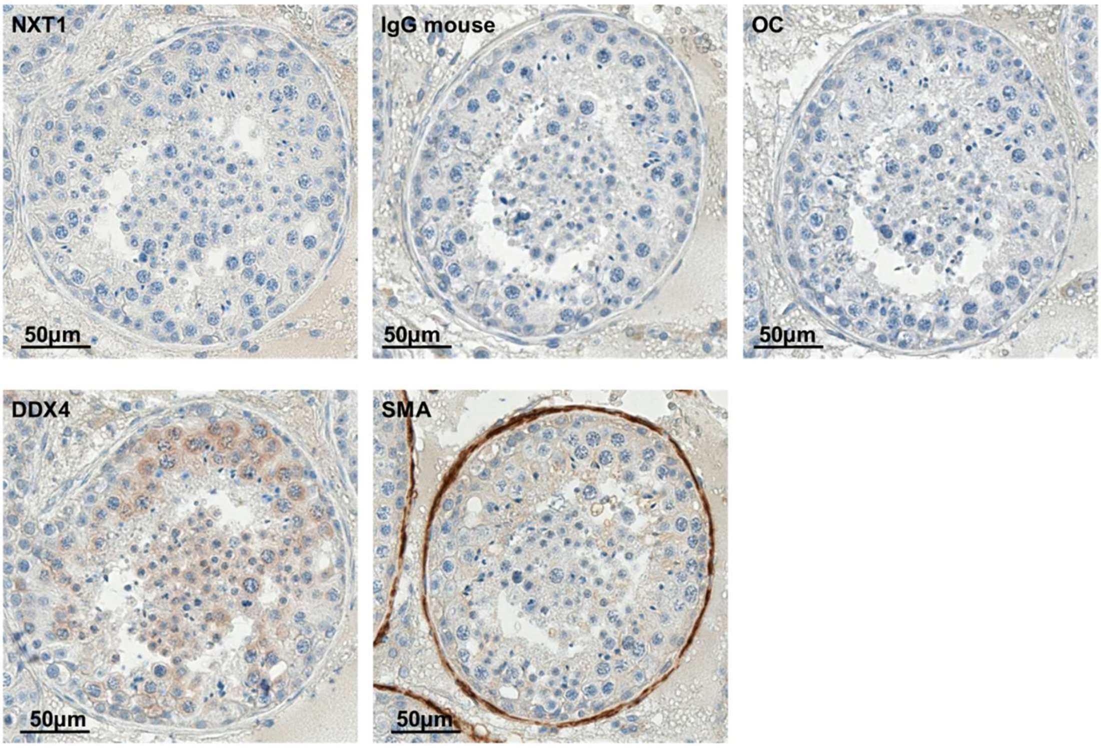

Supplementary Figure 8. No NXT1-specific staining is detectable in human testicular tissue. In testicular tissue of an adult donor with full spermatogenesis no NXT1 specific staining is detectable by immunohistochemical staining with a NXT1-specific monoclonal antibody. Staining for the germ cell marker protein DDX4 and the smooth muscle marker SMA (smouth muscle actin alpha) was used as positive control. IgG mouse: isotype control. OC: omission control (N=3).

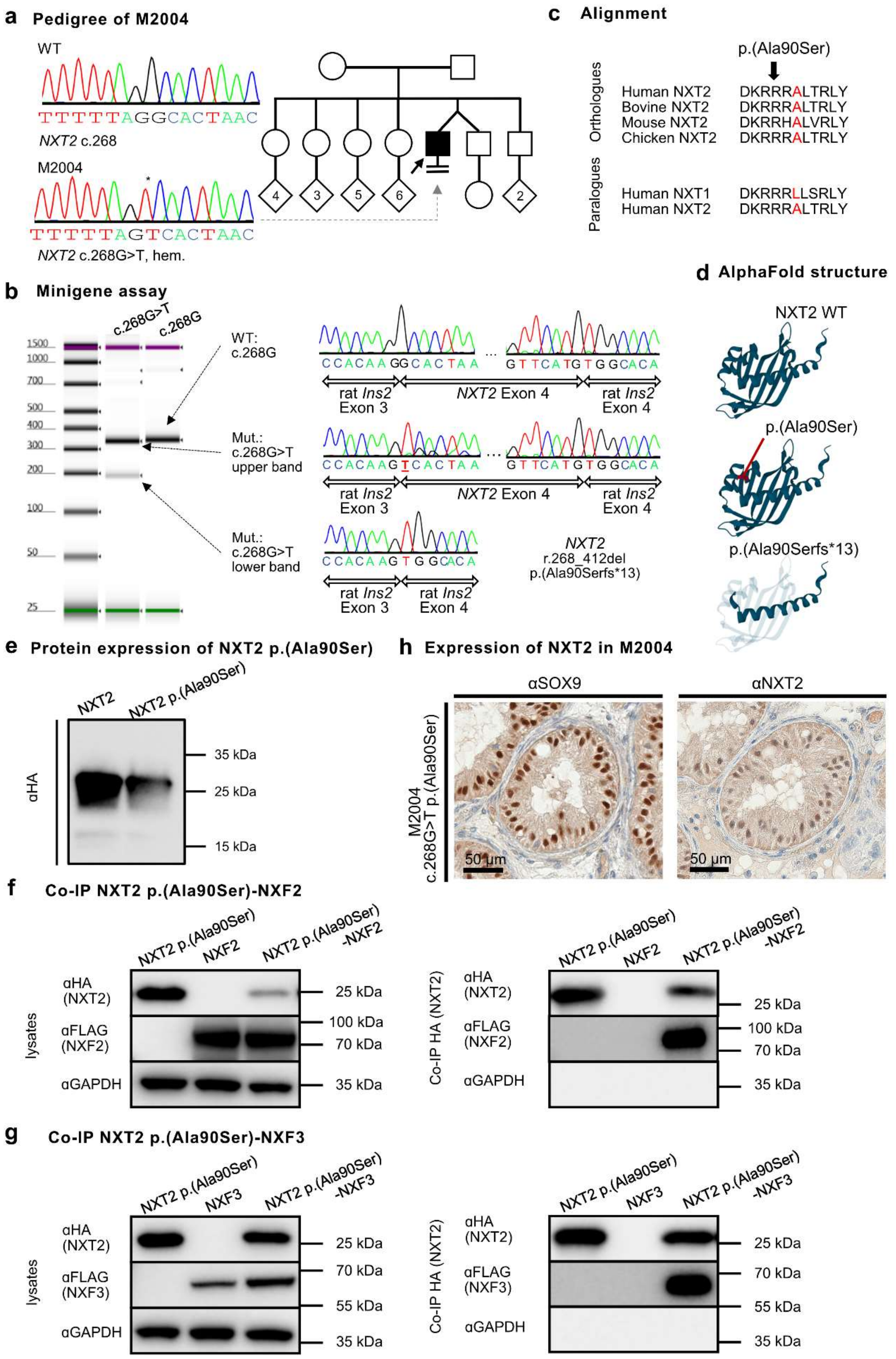
a Pedigree of M2004
WT
TTTTTAGGCACTAAC
NXT2 c.268
M2004
TTTTTAGTCACTAAC
NXT2 c.268G>T, hem.
4
3
5
6
2
b Minigene assay
c.268G>T
c.268G
WT: c.268G
Mut.: c.268G>T upper band
Mut.: c.268G>T lower band
rat Ins2 Exon 3
NXT2 Exon 4
rat Ins2 Exon 4
NXT2 r.268_412del p.(Ala90Serfs*13)
c Alignment
p.(Ala90Ser)
Orthologues
Human NXT2 DKRRRALTRLY
Bovine NXT2 DKRRRALTRLY
Mouse NXT2 DKRRHALVRLY
Chicken NXT2 DKRRRALTRLY
Paralogues
Human NXT1 DKRRRLLSRLY
Human NXT2 DKRRRALTRLY
d AlphaFold structure
NXT2 WT
p.(Ala90Ser)
p.(Ala90Serfs*13)
e Protein expression of NXT2 p.(Ala90Ser)
NXT2
NXT2 p.(Ala90Ser)
αHA
35 kDa
25 kDa
15 kDa
h Expression of NXT2 in M2004
αSOX9
αNXT2
M2004 c.268G>T p.(Ala90Ser)
50 µm
f Co-IP NXT2 p.(Ala90Ser)-NXF2
NXT2 p.(Ala90Ser)
NXF2
NXT2 p.(Ala90Ser) -NXF2
lysates
Co-IP HA (NXT2)
αHA (NXT2)
αFLAG (NXF2)
αGAPDH
25 kDa
100 kDa
70 kDa
35 kDa
g Co-IP NXT2 p.(Ala90Ser)-NXF3
NXT2 p.(Ala90Ser)
NXF3
NXT2 p.(Ala90Ser) -NXF3
αFLAG (NXF3)
55 kDa

Supplementary Figure 9. The *NXT2* missense variant c.268G>T p.(Ala90Ser) in M2004 has an impact on splicing but no identified effect on the protein level. a. M2004 is positive for the hemizygous single nucleotide substitution c.268G>T in *NXT2* affecting the most 5' nucleotide of exon four. DNA samples of other family members were not available for co-segregation analysis. b. In a minigene assay, the variant c.268G>T in *NXT2* leads to a skipping of exon 4 in a minor proportion of transcripts *in vitro*, resulting in a frameshift [r.268_412del p.(Ala90Serfs*13)]. c. The affected alanine residue is conserved in orthologs of NXT2 across bovine, mouse and chicken. In the paralog human protein NXT1, the amino acid is not preserved. d. AlphaFold2 predictions show wildtype, mutant (substituted residue = red) and truncated (loss of 107 amino acids caused by exon skipping) NXT2 3D structures. e, Western blot of overexpressed wildtype and mutant *NXT2* p.(Ala90Ser) demonstrated no effect of the amino acid substitution on protein stability. f, When overexpressed in HEK cells, NXT2 p.(Ala90Ser) and NXF2 are detectable (~26 kDa for NXT2 and ~72 kDa for NXF2) in a Western blot of cell lysates (left). The NXT2 p.(Ala90Ser) variant does not impair binding to NXF2 as shown by Co-IP experiments (right). g. Binding of NXT2 p.(Ala90Ser) to NXF3 is also not affected as shown by Co-IP. Western blots demonstrate representative results of at least three replicates (N=3). Source data are provided as a Source Data file. h. Immunohistochemical staining of NXT2 in M2004 compared to control tissue demonstrated that NXT2 is still expressed in SOX9-positive Sertoli cells (N=3).

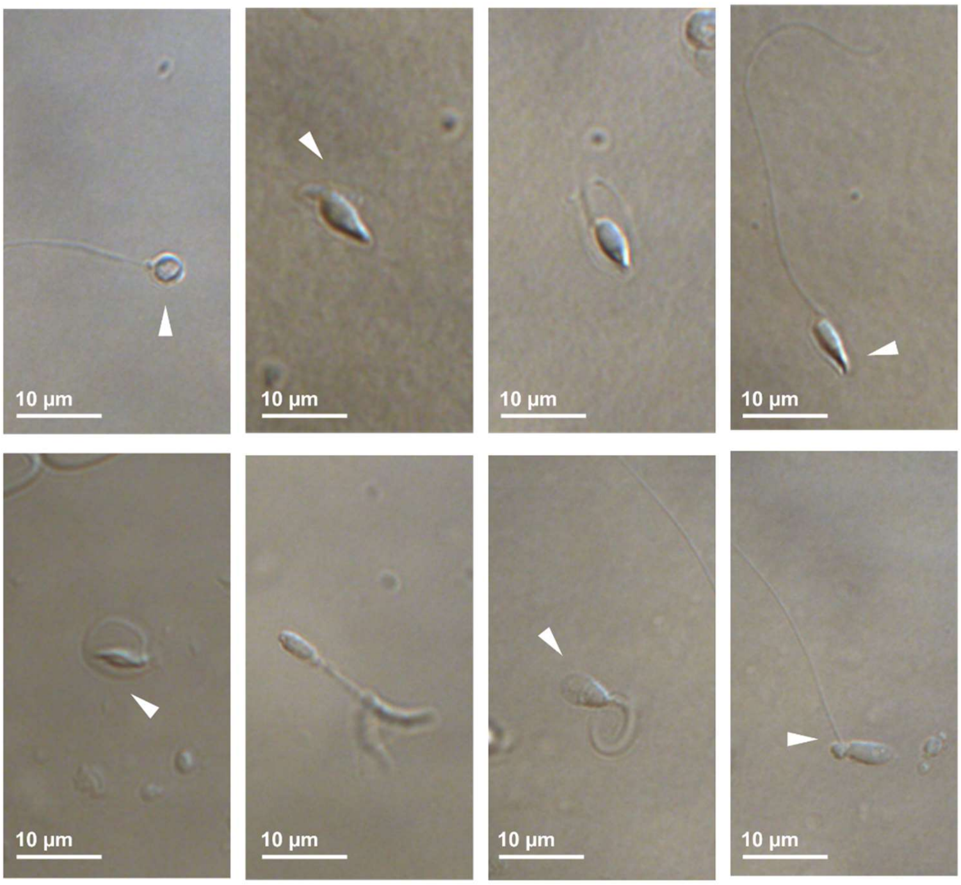

Supplementary Figure 10. DIC microscopy of sperm from M2799 with LoF variant in NXF3. Abnormal sperm shapes identified in M2799 include amorphous or round heads, bent midpiece and a short, bent or tightly curled tail (N=3).

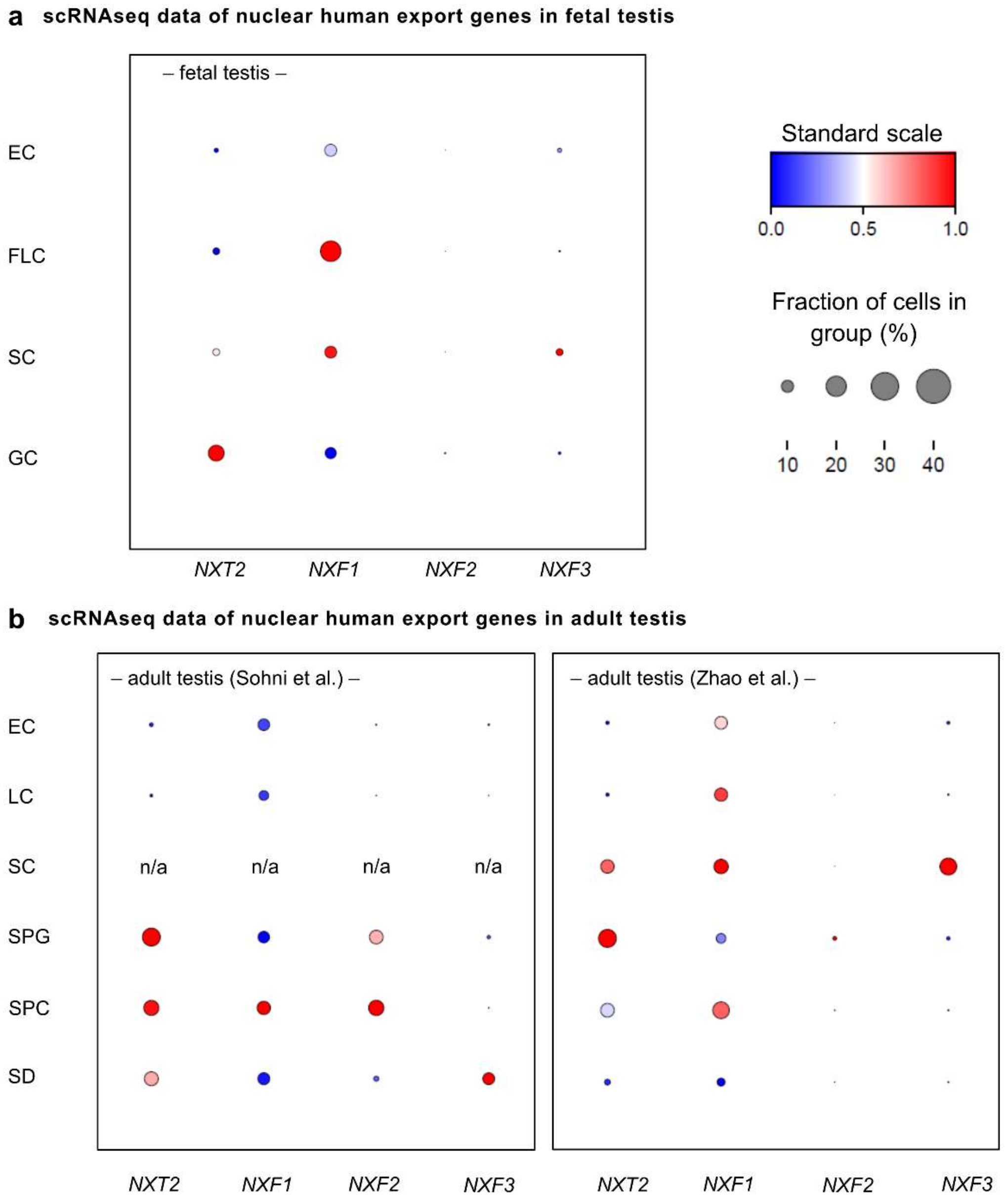

Supplementary Figure 11. scRNA-seq shows expression of nuclear export factor proteins in fetal male germ cells and adult testis. a. scRNA-seq dataset of fetal male germ cells[2] was analyzed via smart-DB[3]. *NXT2* reveals a strong expression in germ cells. *NXF1* is mainly expressed in fetal Leydig and in Sertoli cells. *NXF2* is generally weakly expressed and *NXF3* is expressed in fetal Sertoli but not in germ cells. b. In the adult testis, different scRNA-seq datasets[4,5] showed different expression patterns: Sohni et al. show *NXT2* expression mainly in spermatogonia, but also in spermatocytes and spermatids[4]. *NXF1* is ubiquitously expressed, *NXF2* is expressed in spermatogonia and spermatocytes and *NXF3* is mainly expressed in spermatids (left)[4]. In the dataset of Zhao et al. (right)[5] *NXT2*, *NXF1* and *NXF3* are also expressed in Sertoli cells (EC: endothelial cells; FLC: fetal Leydig cells; LC: Leydig cells; SC: Sertoli cells; GC: germ cells; SPG: spermatogonia; SPC: spermatocytes; SD: spermatids).

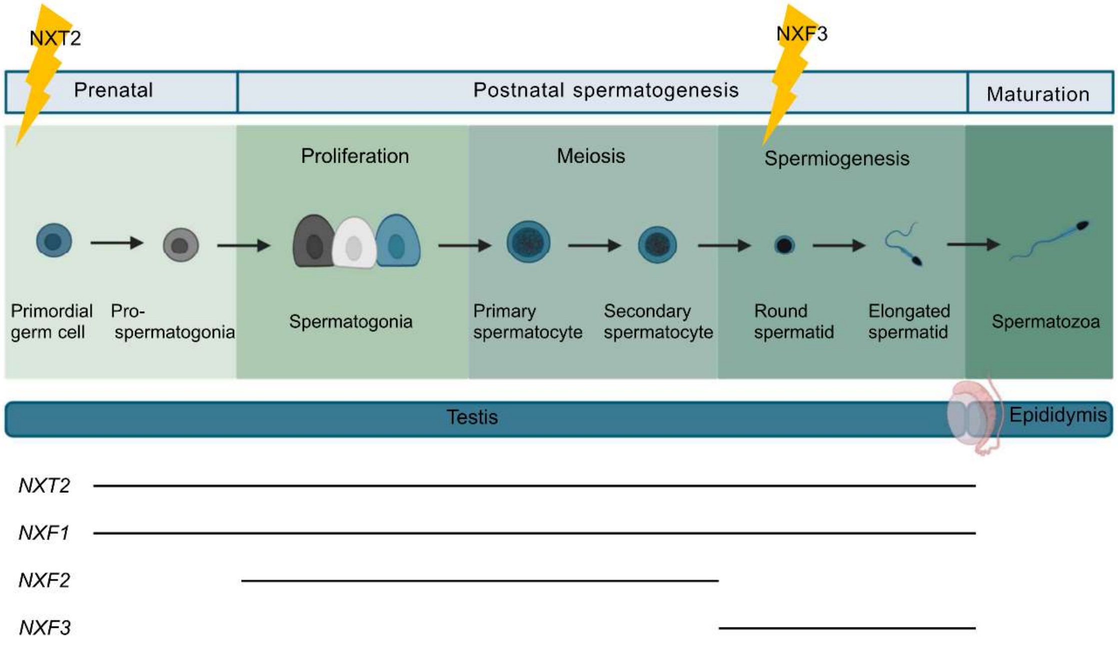

Supplementary Figure 12. Schematic illustration of germ cell development with temporal expression profiles of nuclear export factor family genes. Spermatogenesis can be subdivided into three main stages: Spermatogonial stem cells undergo mitosis to form primary spermatocytes, which then enter meiosis. During meiosis primary spermatocytes divide twice to produce haploid secondary spermatocytes. Secondary spermatocytes develop to round spermatids. In spermiogenesis, round spermatids undergo structural changes, including condensation of the nucleus, development of sperm flagellum and acrosome, transforming into mature sperm. The loss of NXT2 leads to absence of spermatogonia, while impaired NXF3 function impacts the later stages of germ cell development, resulting in abnormal sperm morphology and reduced sperm count. Created in BioRender. Stallmeyer, B. (2025) https://BioRender.com/x7s7j43.

Supplementary References

1. Oud, M. S. *et al.* A de novo paradigm for male infertility. *Nature Communications* **13**, 154 (2022).

2. Garcia-Alonso, L. *et al.* Single-cell roadmap of human gonadal development. *Nature* **607**, 540–547 (2022).

3. Liu, Z. *et al.* SMARTdb: An Integrated Database for Exploring Single-cell Multi-omics Data of Reproductive Medicine. *Genomics, Proteomics & Bioinformatics* **38**, e102870 (2024).

4. Sohni, A. *et al.* The Neonatal and Adult Human Testis Defined at the Single-Cell Level. *Cell reports* **26**, 1501-1517.e4 (2019).

5. Zhao, L. Y. *et al.* Single-cell analysis of developing and azoospermia human testicles reveals central role of Sertoli cells. *Nature communications* **11**, 5683 (2020).

Source Data Supplementary Figures

Source Data Supplementary Figure 1a

αHA:

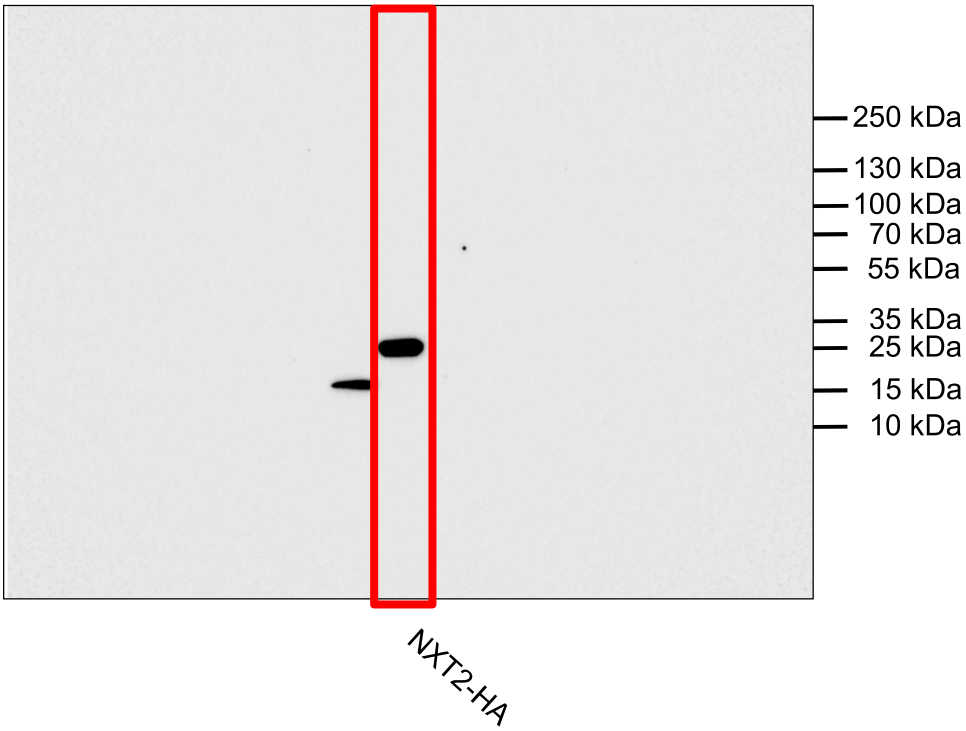

αNXT2:

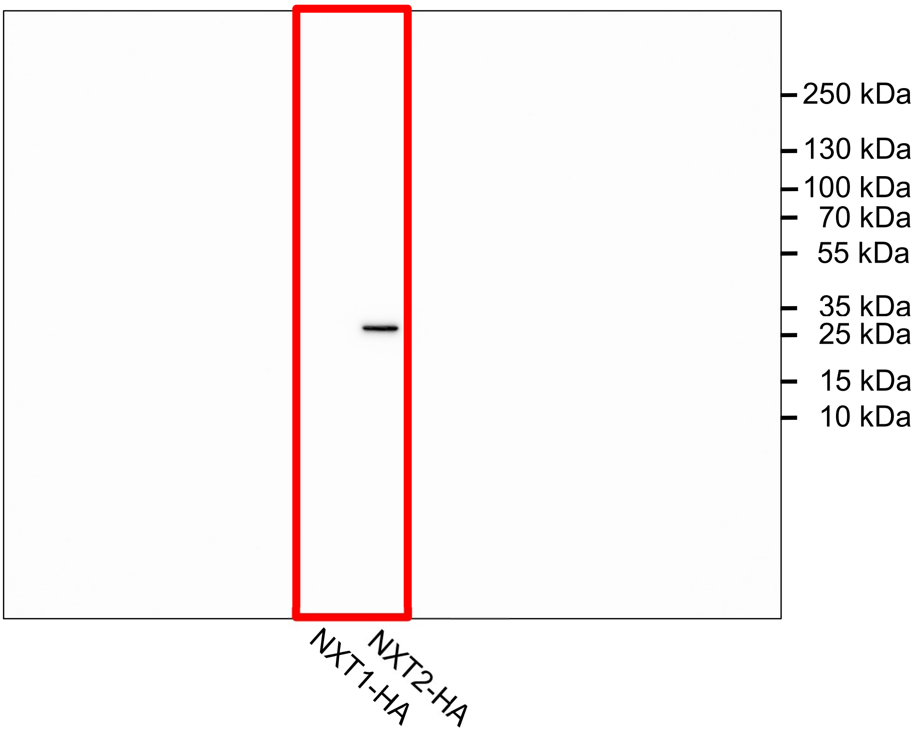

αNXT2:

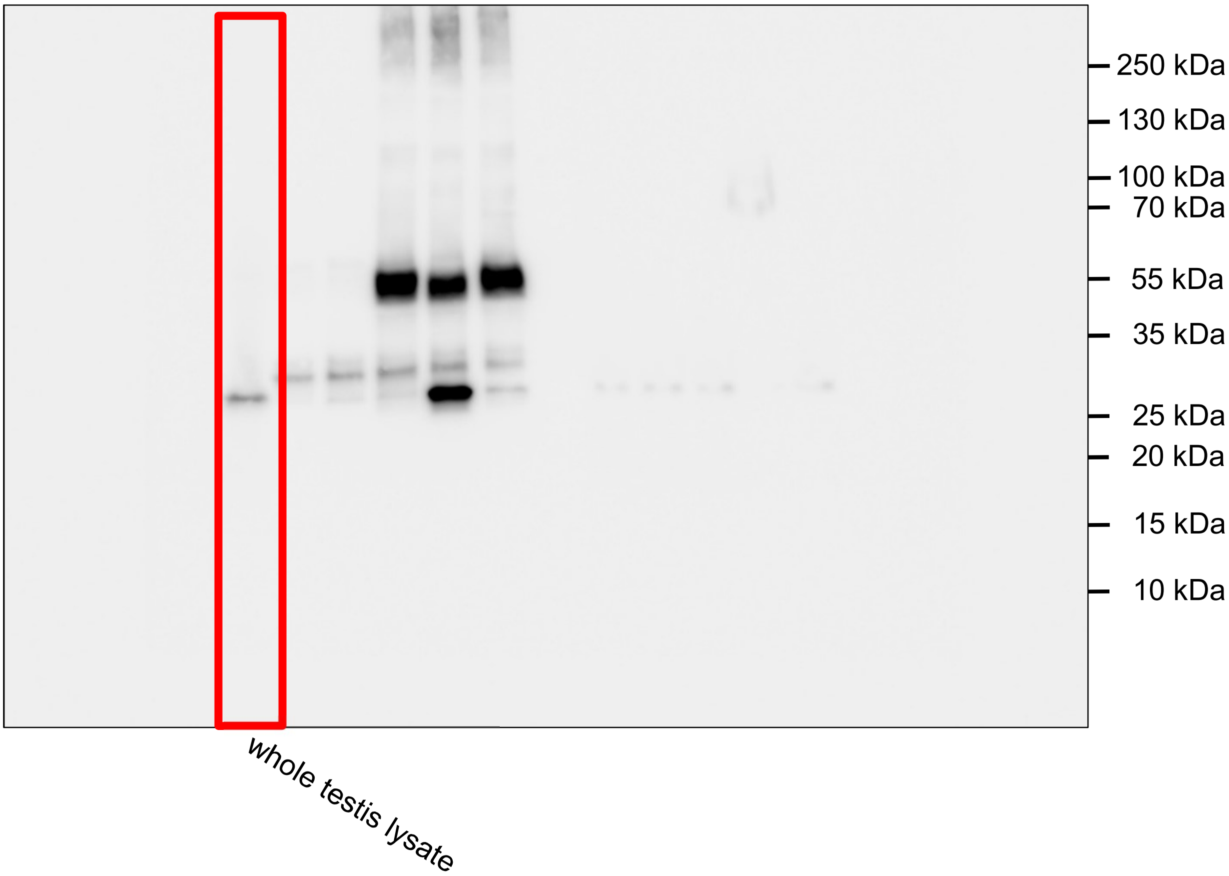
250 kDa
130 kDa
100 kDa
70 kDa
55 kDa
35 kDa
25 kDa
20 kDa
15 kDa
10 kDa
whole testis lysate

Source Data Supplementary Figure 1b

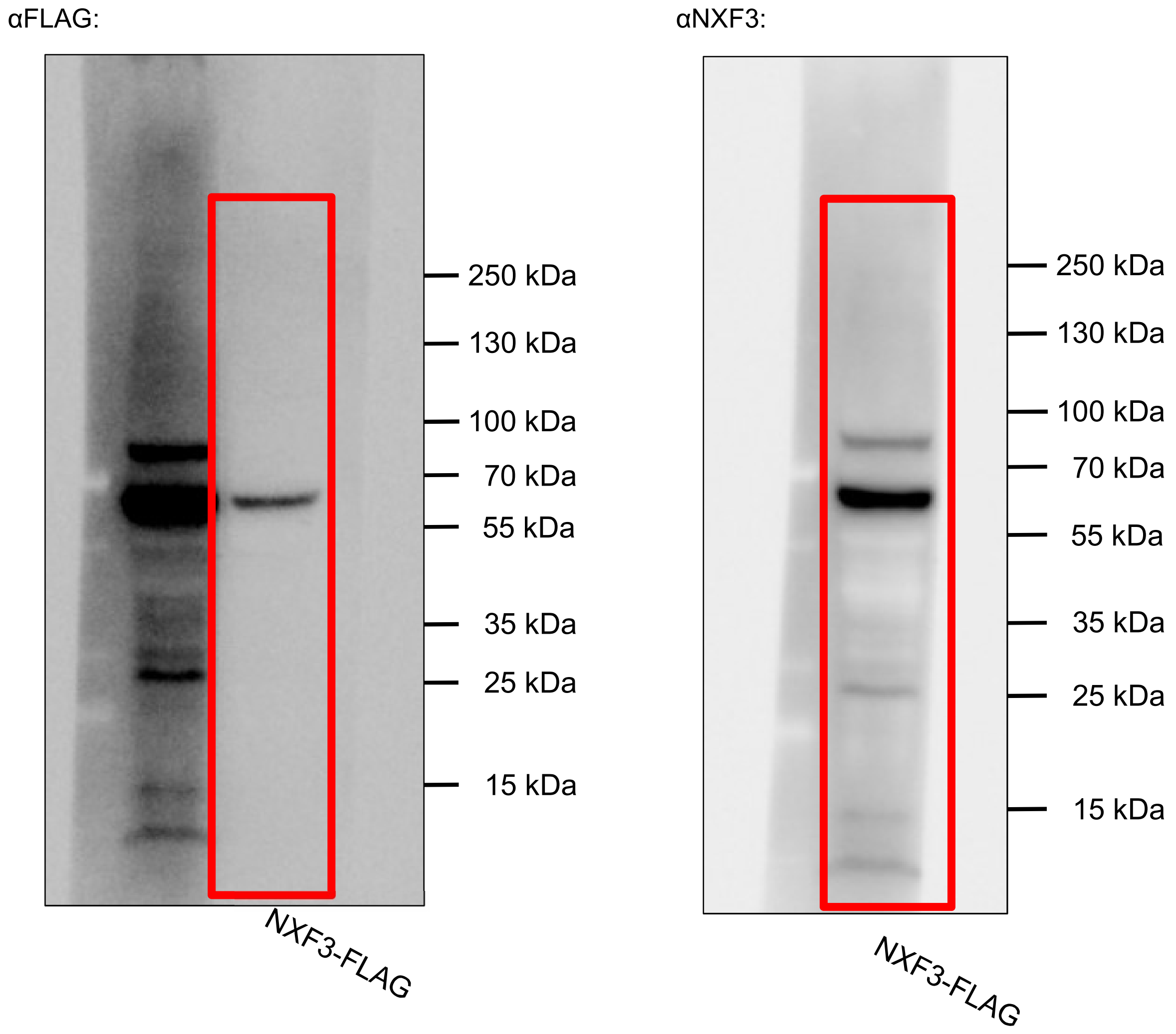

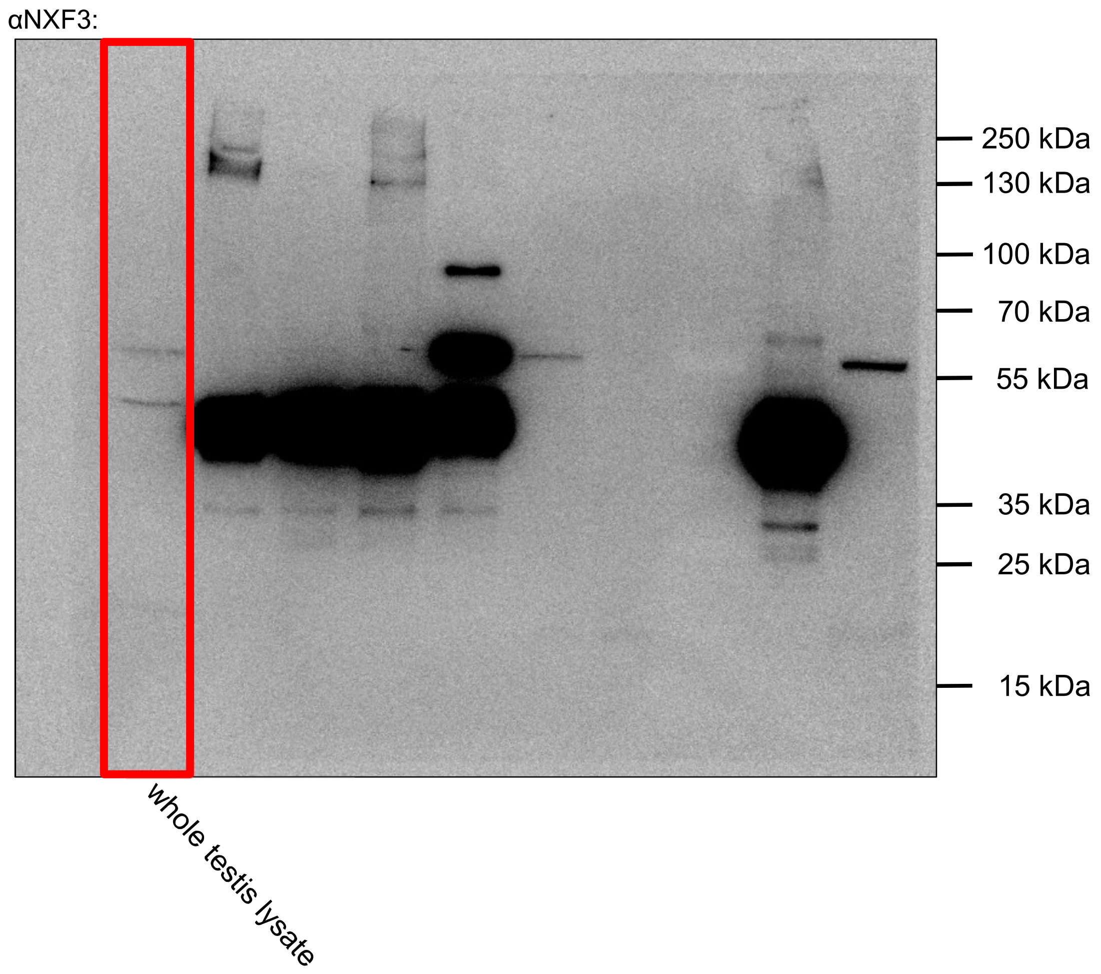
αNXF3:
250 kDa
130 kDa
100 kDa
70 kDa
55 kDa
35 kDa
25 kDa
15 kDa
whole testis lysate

Source Data Supplementary Figure 1c

αHA:

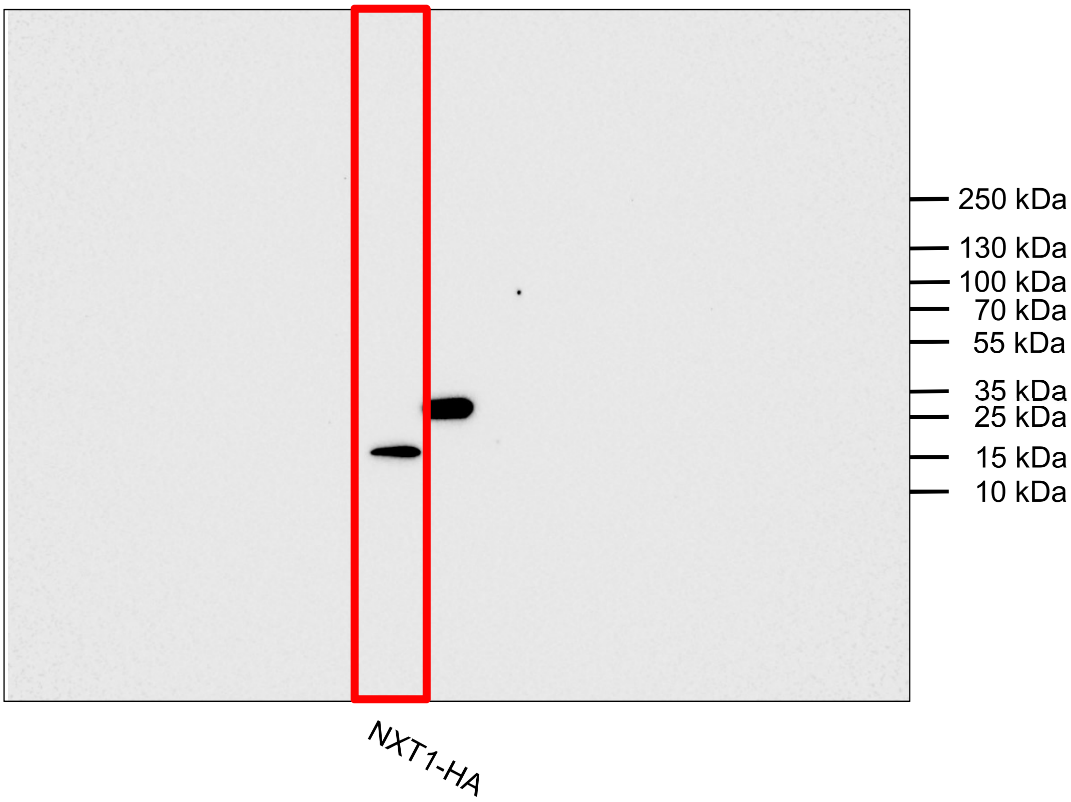

αNXT1:

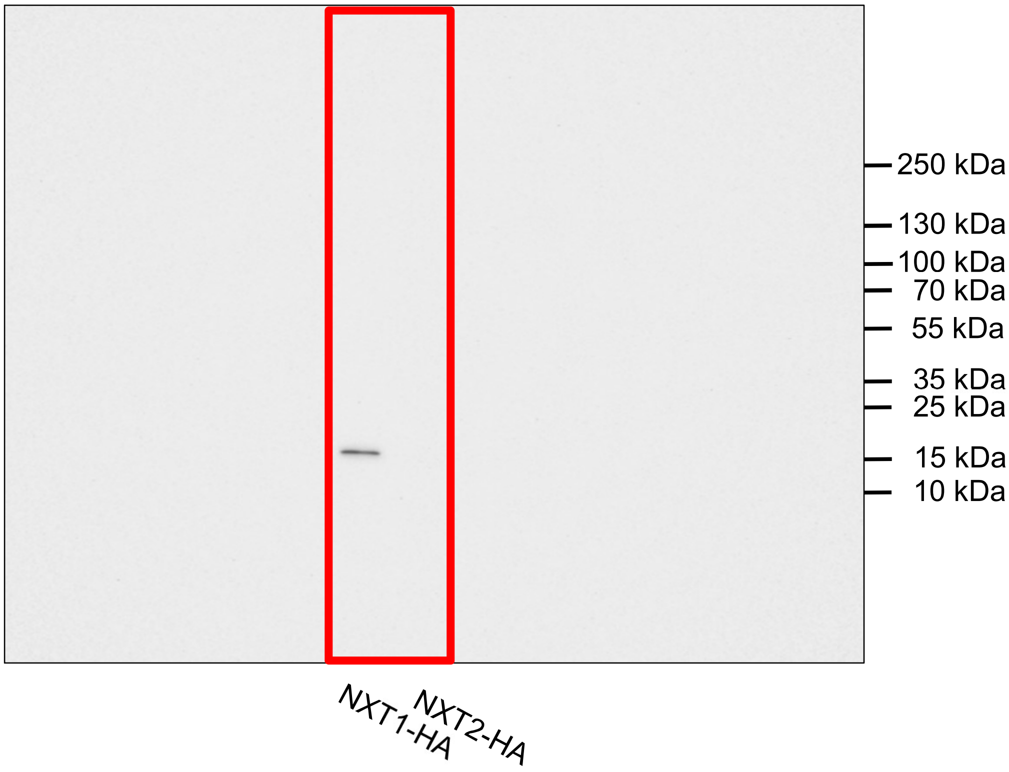

Source Data Supplementary Figure 2a

αNXT2:

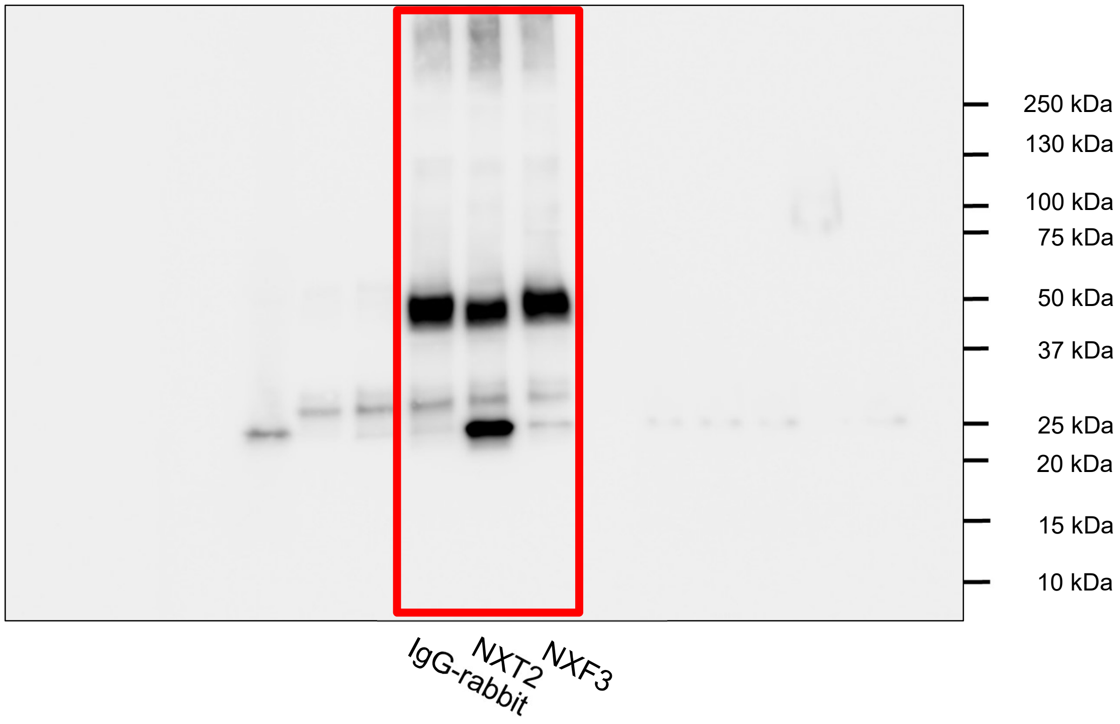

Source Data Supplementary Figure 2b

αNXF3:

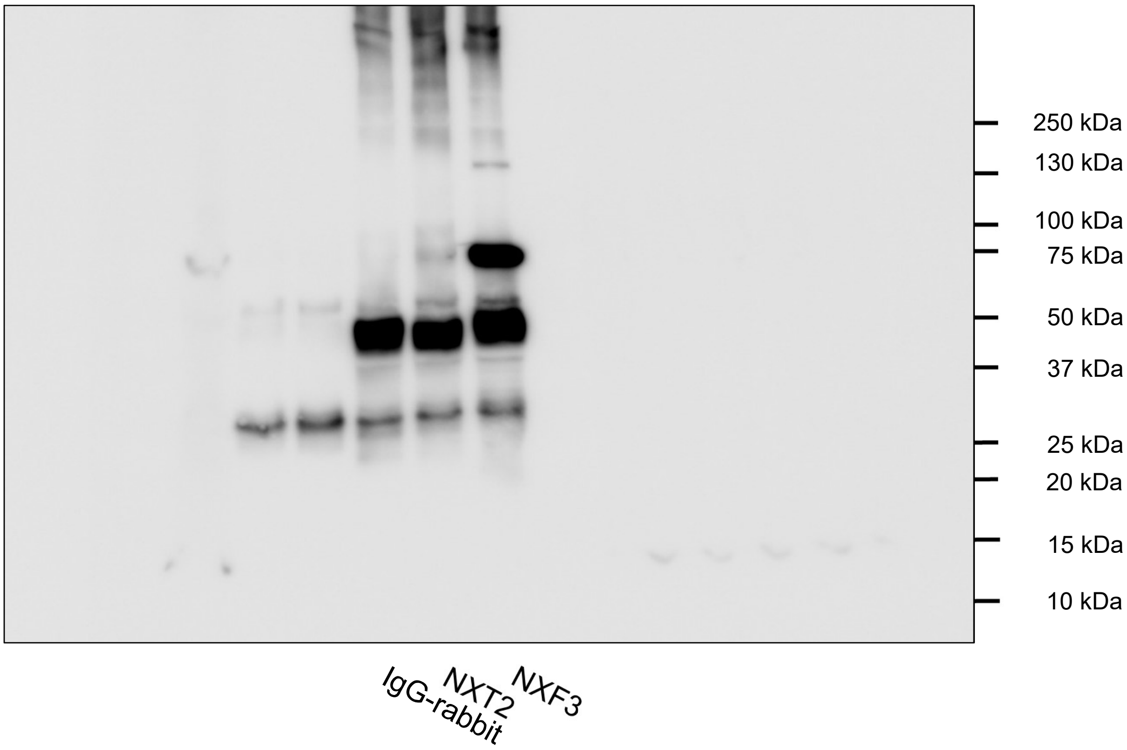

Source Data Supplementary Figure 2c

αNXT1:

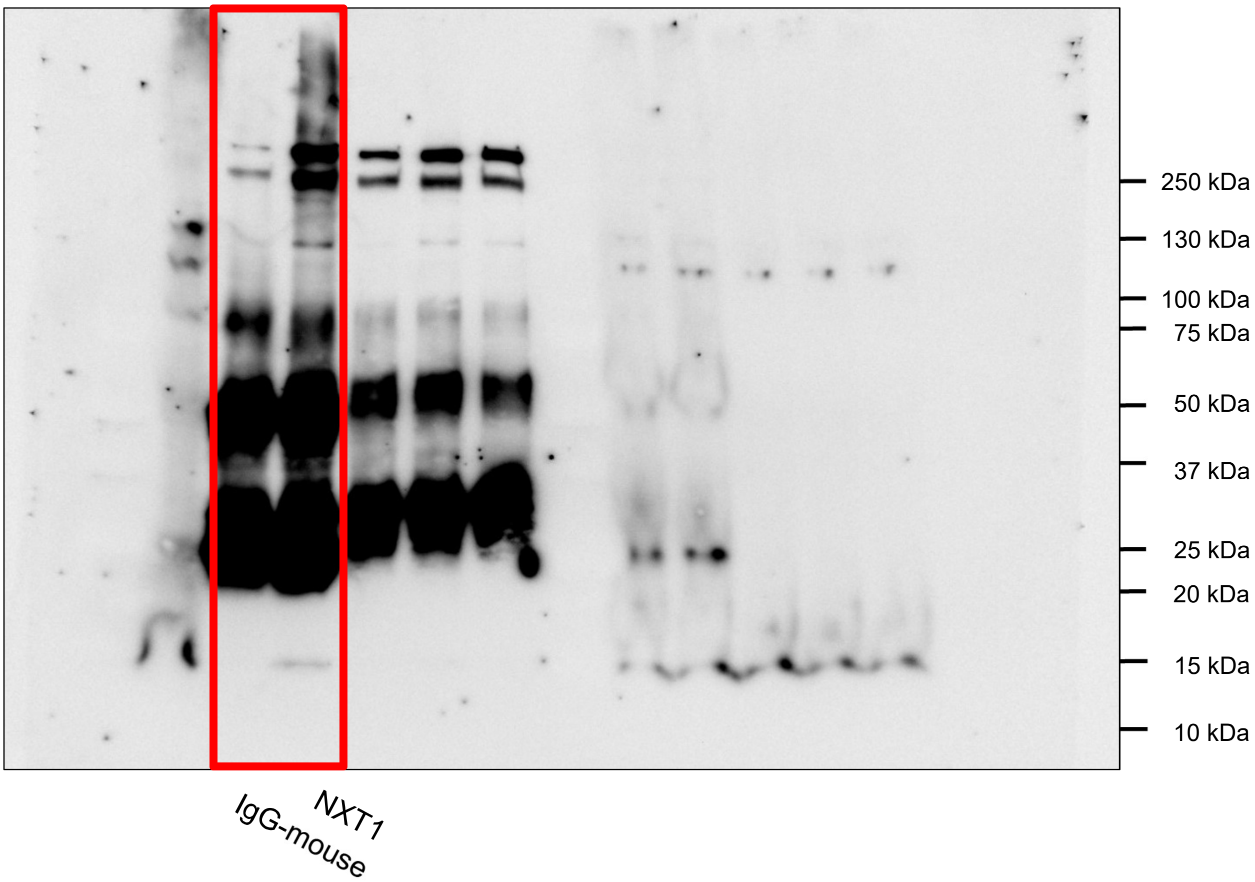

Source Data Supplementary Figure 5a

αFLAG:

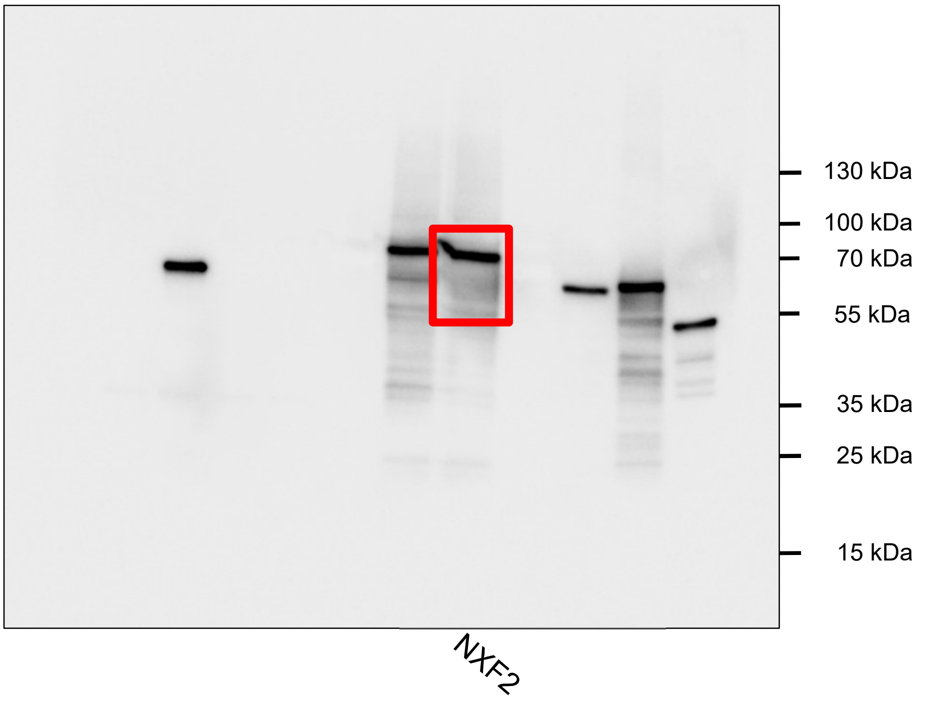

αFLAG:

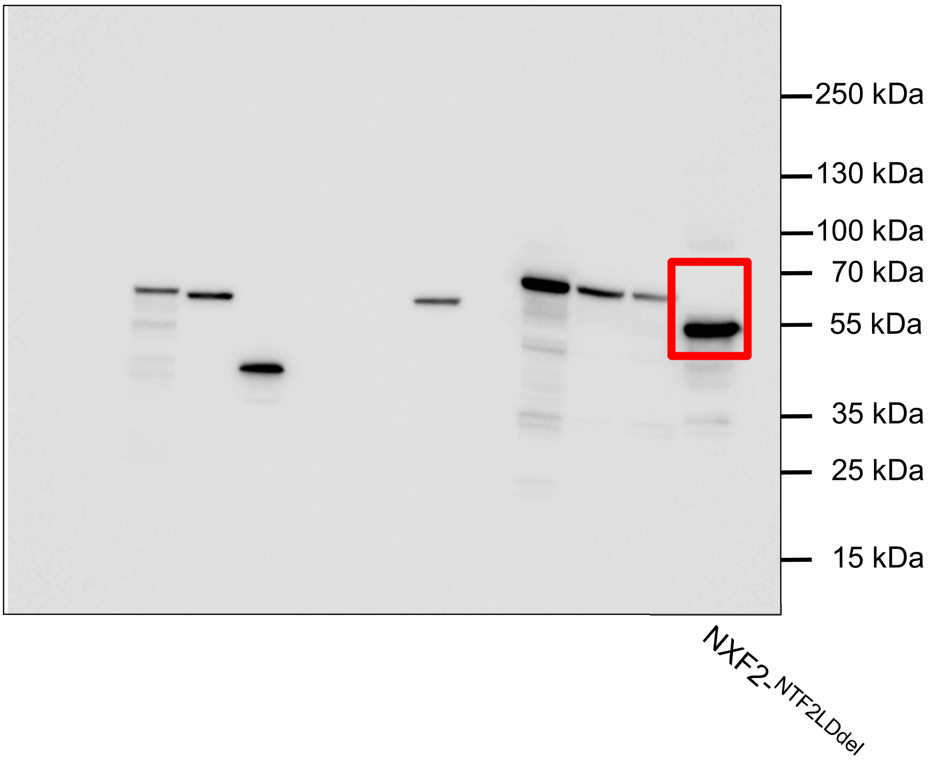

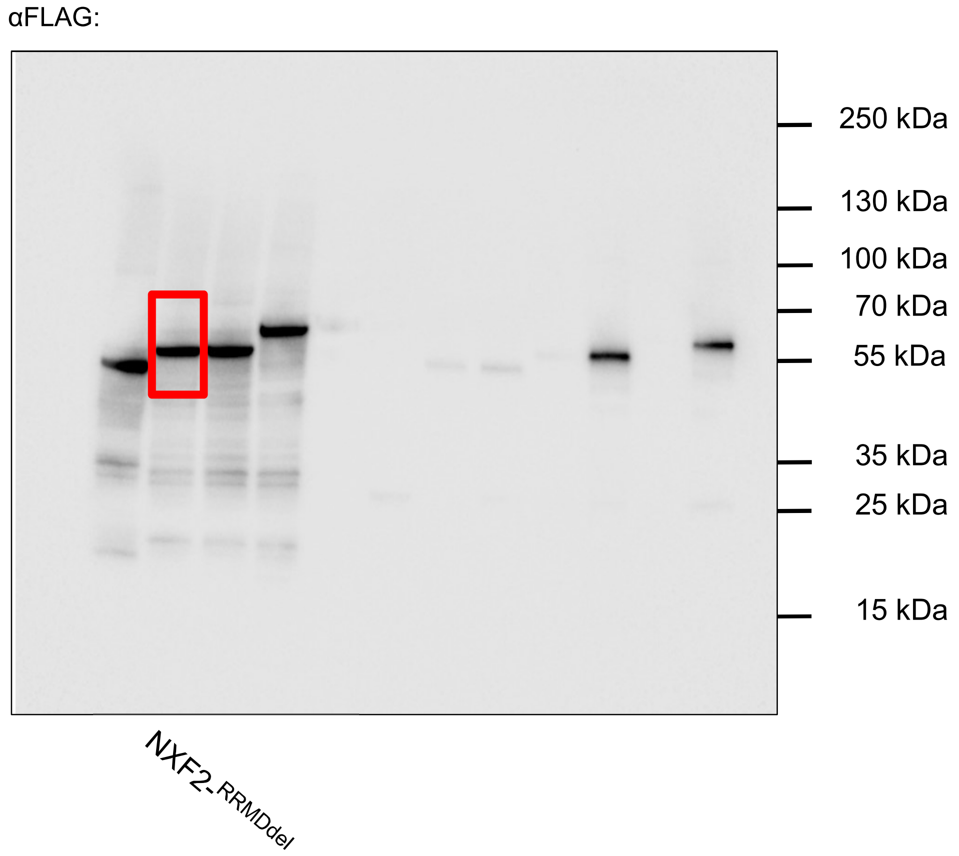
αFLAG:
250 kDa
130 kDa
100 kDa
70 kDa
55 kDa
35 kDa
25 kDa
15 kDa
NXF2-RRMDdel

Source Data Supplementary Figure 5b

αFLAG:

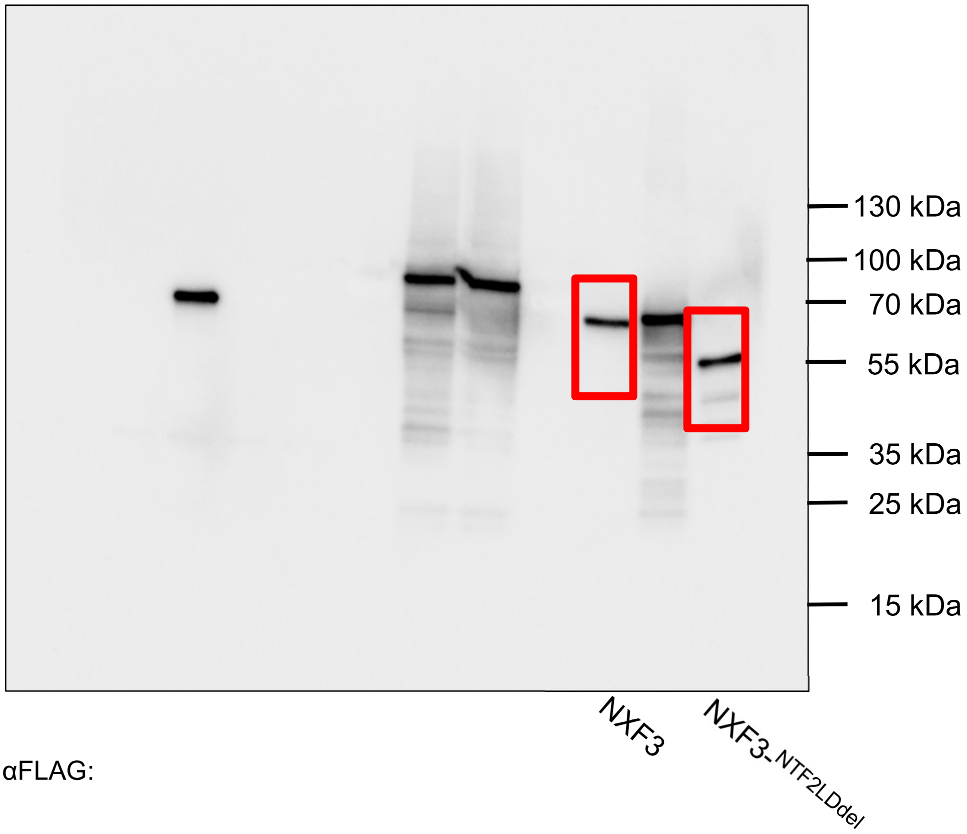

αFLAG:

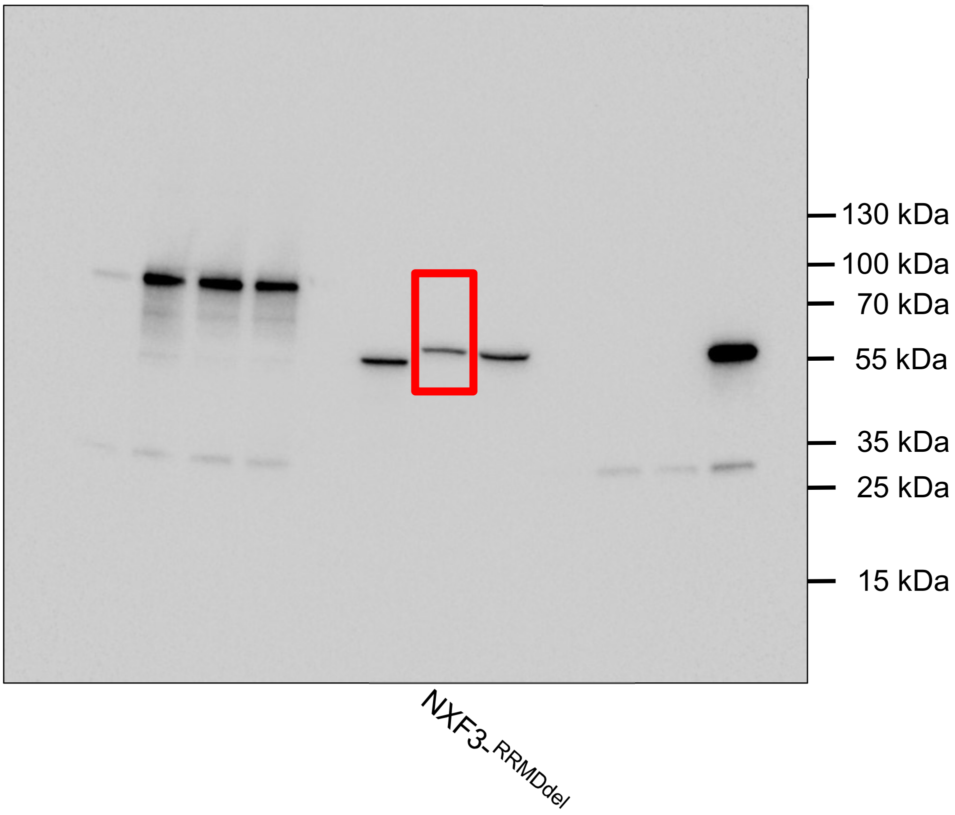

Source Data Supplementary Figure 6b

Lysates

αHA:

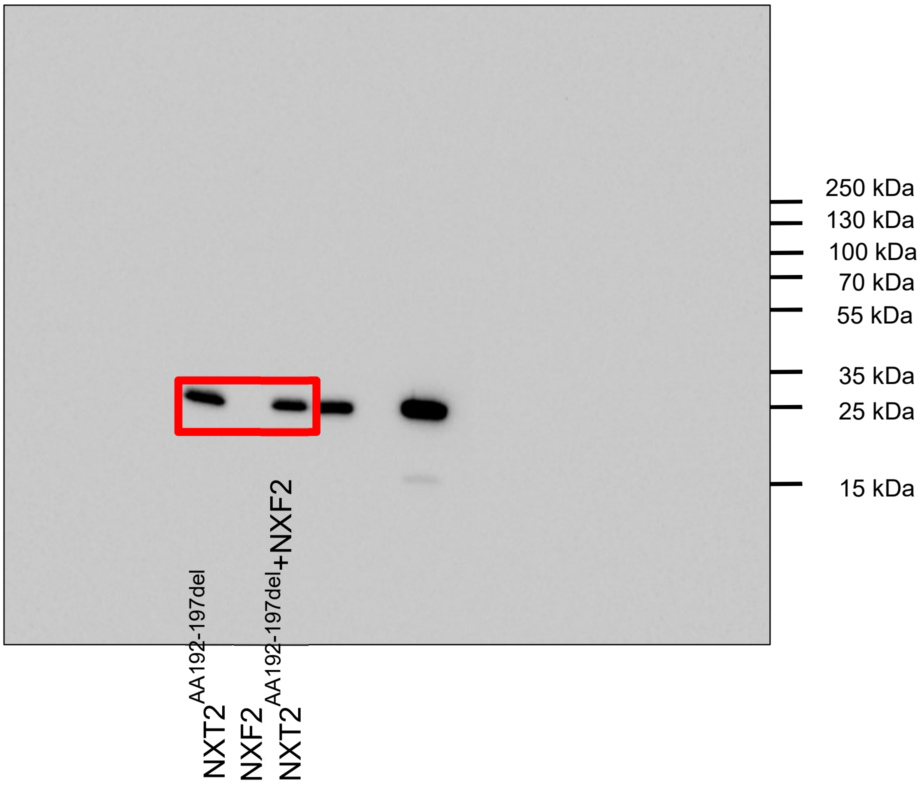

αFLAG:

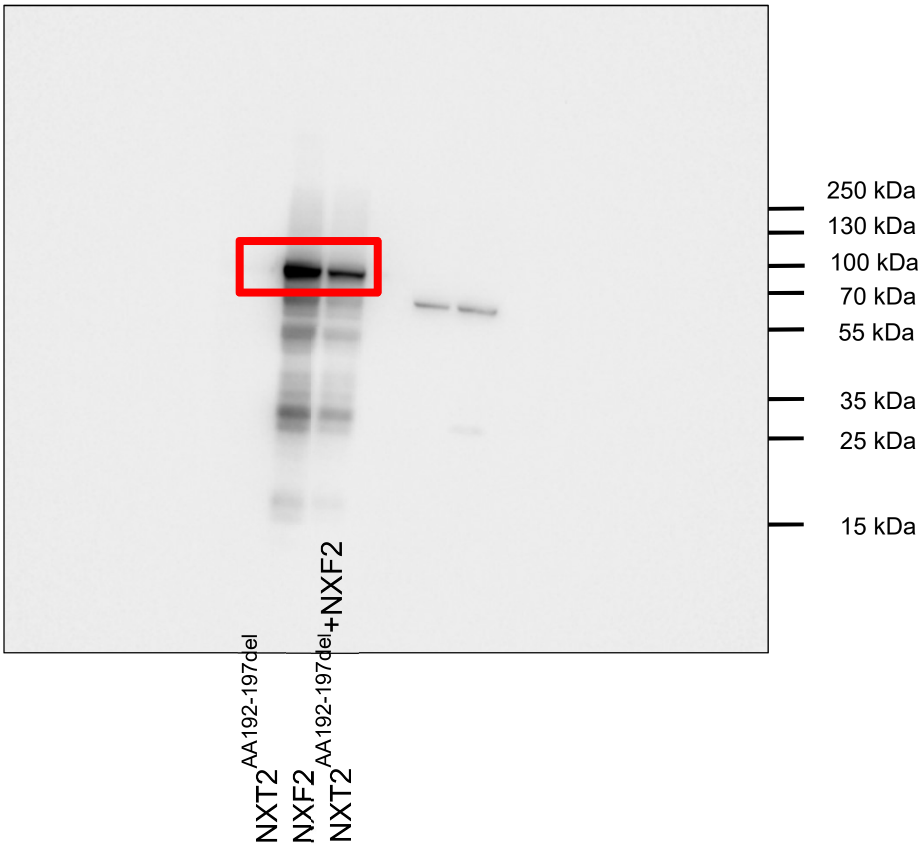

αGAPDH:

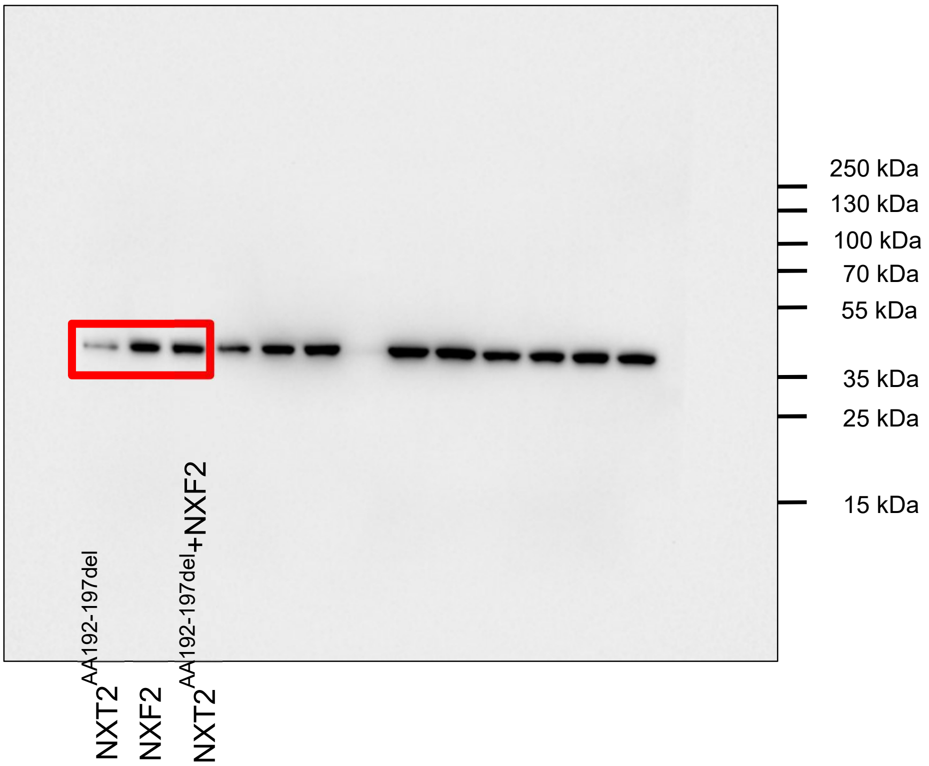

Co-IP

αHA:

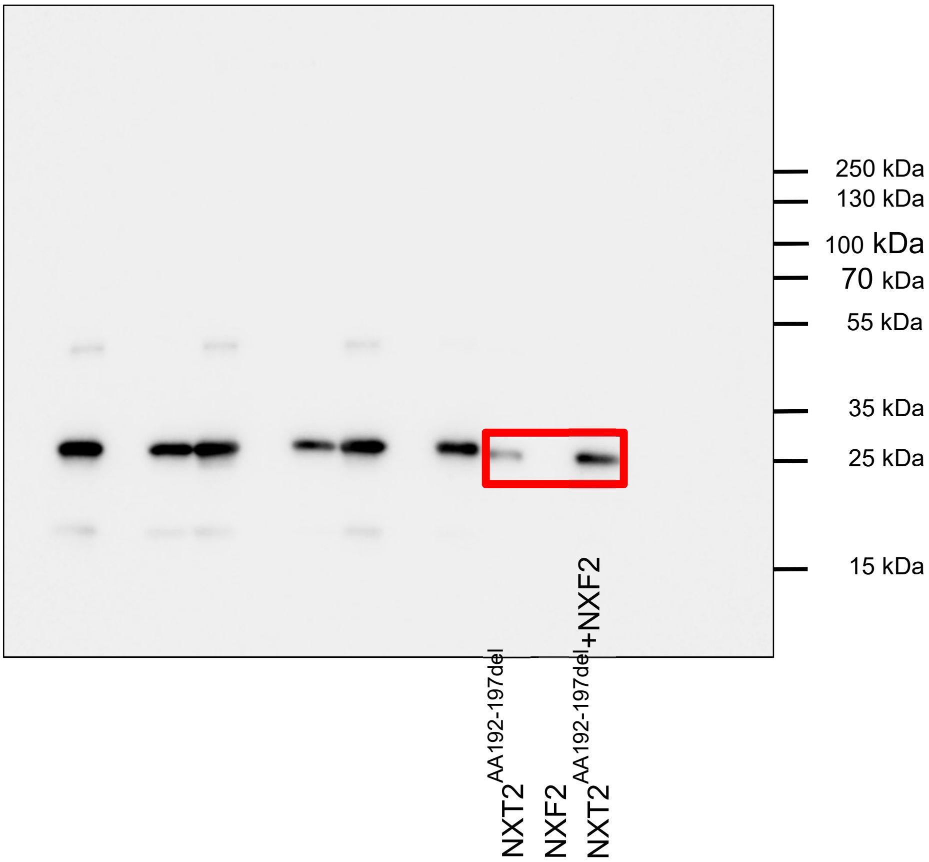

αFLAG:

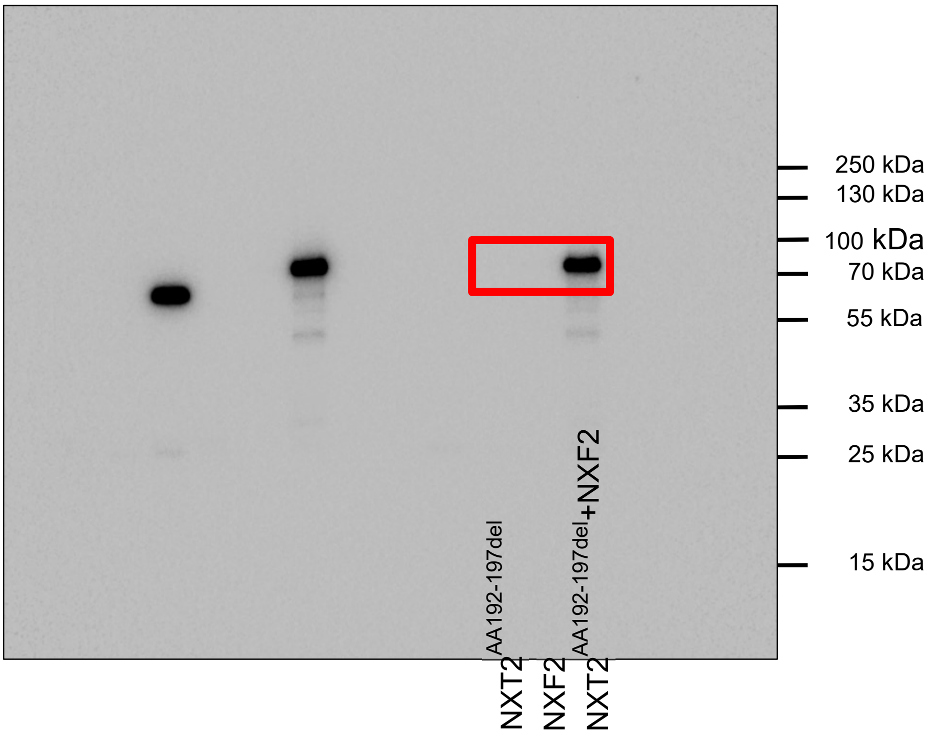

αGAPDH:

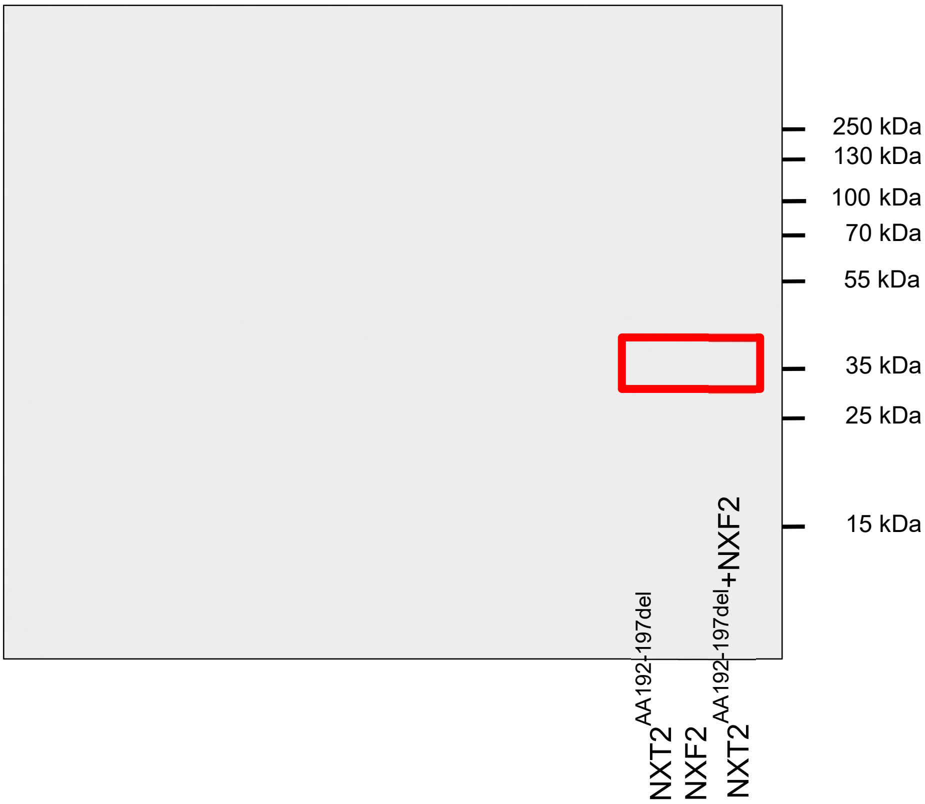

Source Data Supplementary Figure 6c

Lysates

αHA:

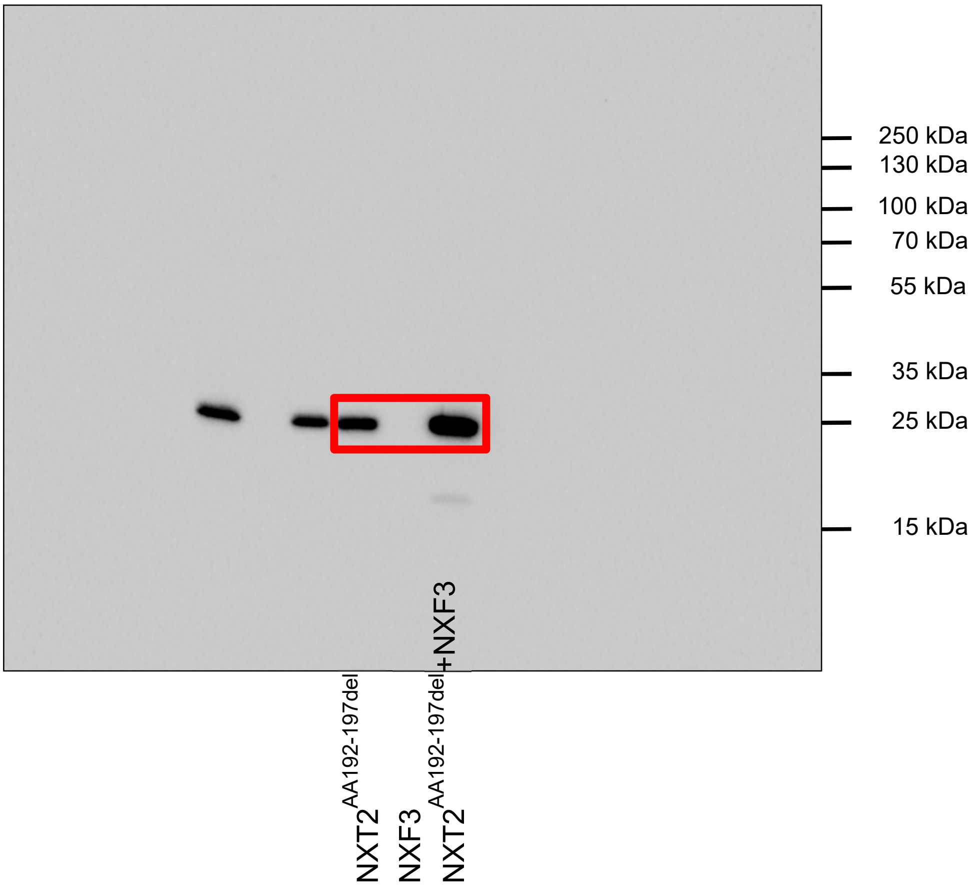

αFLAG:

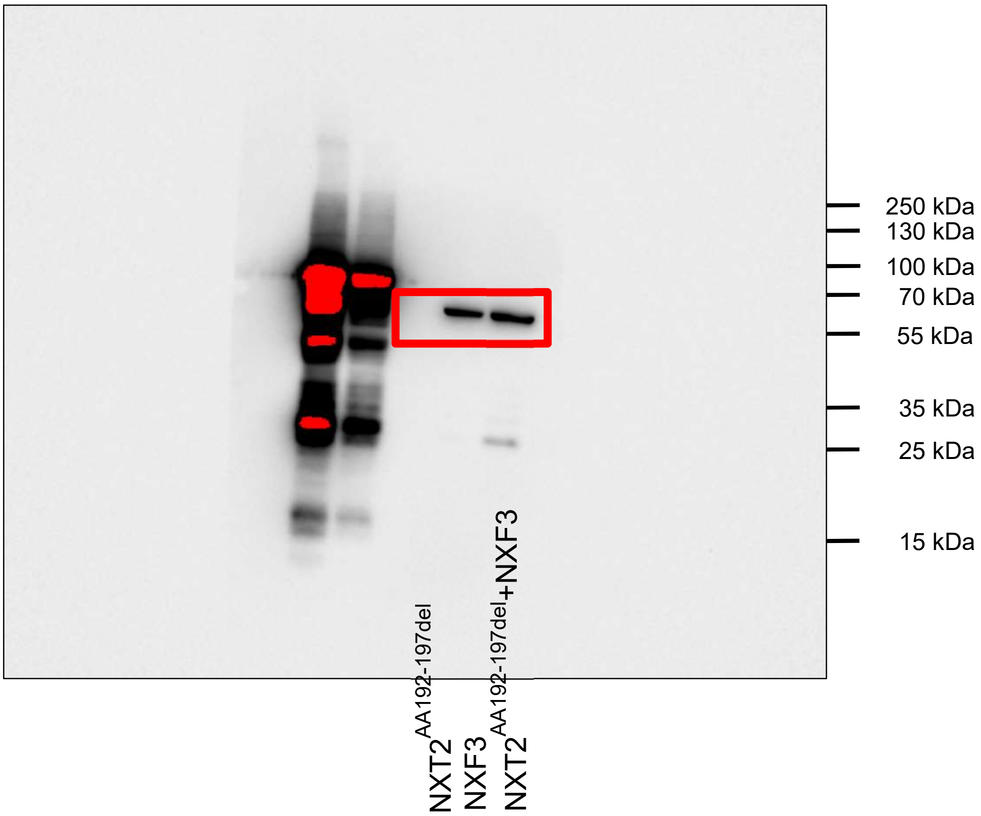

αGAPDH:

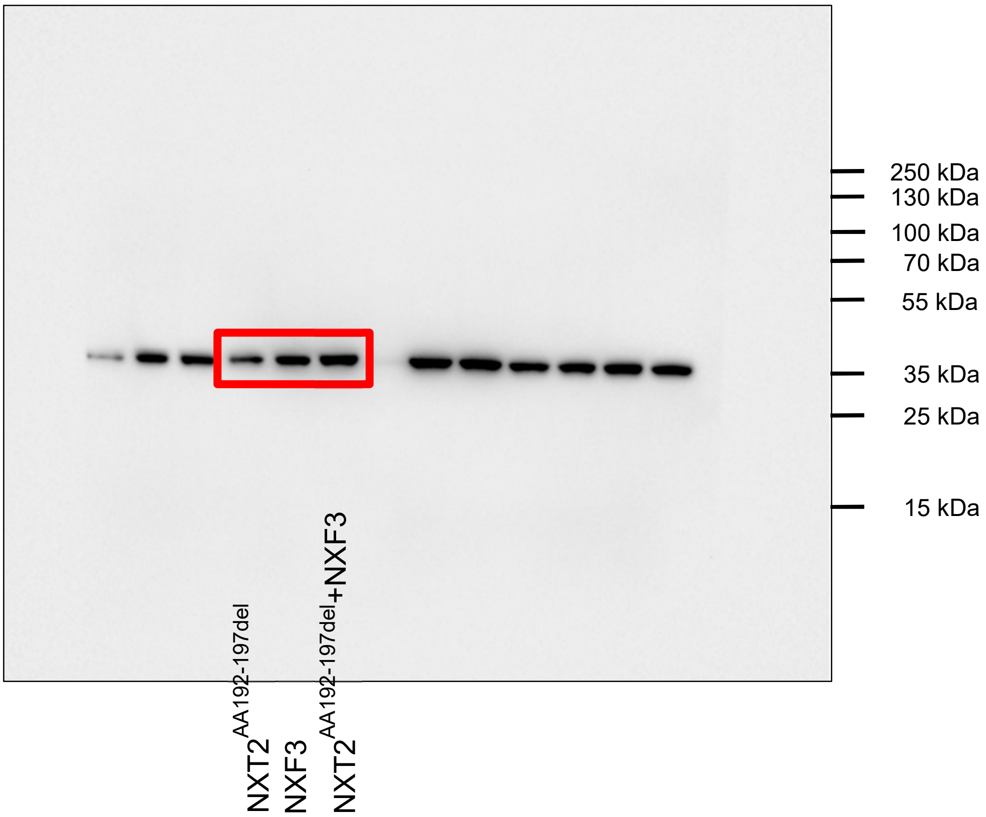

Co-IP

αHA:

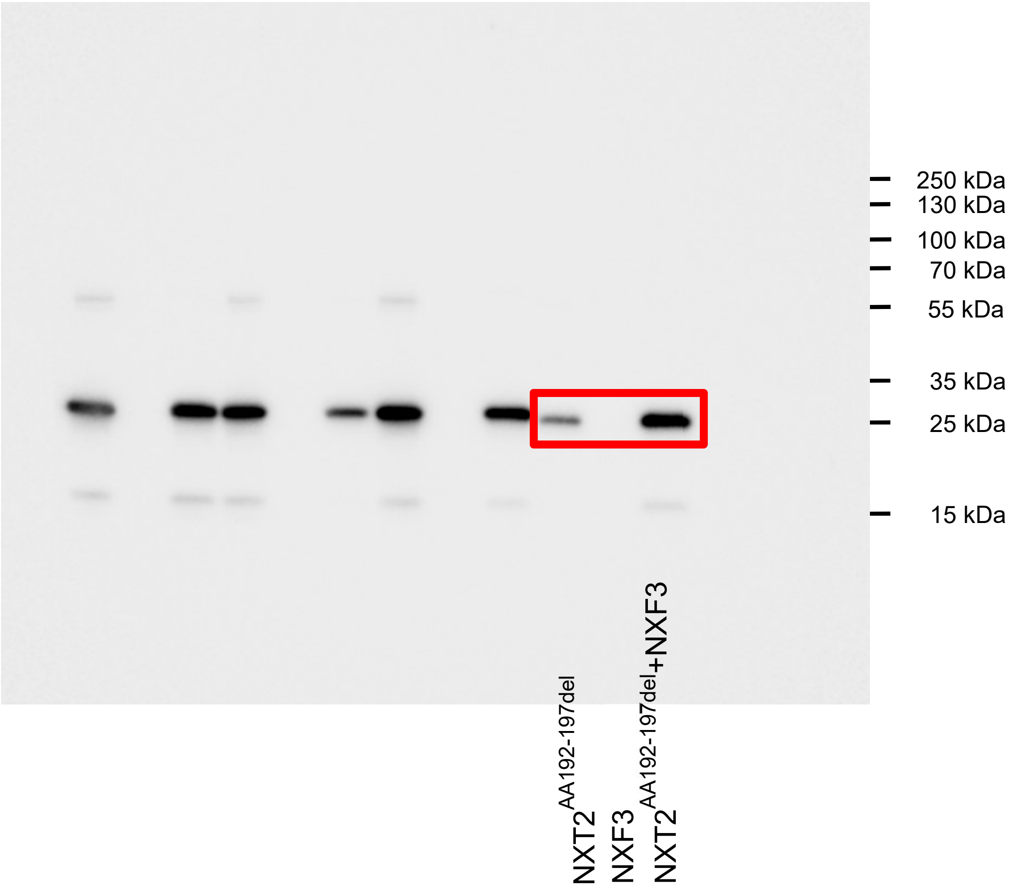

αFLAG:

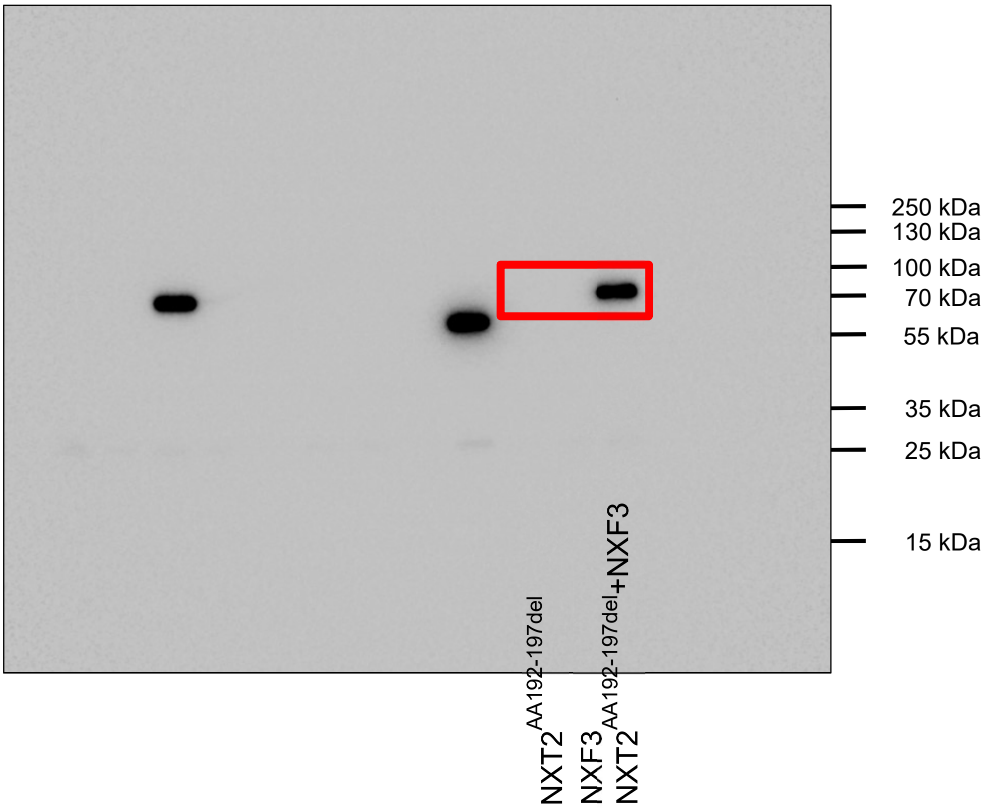

αGAPDH:

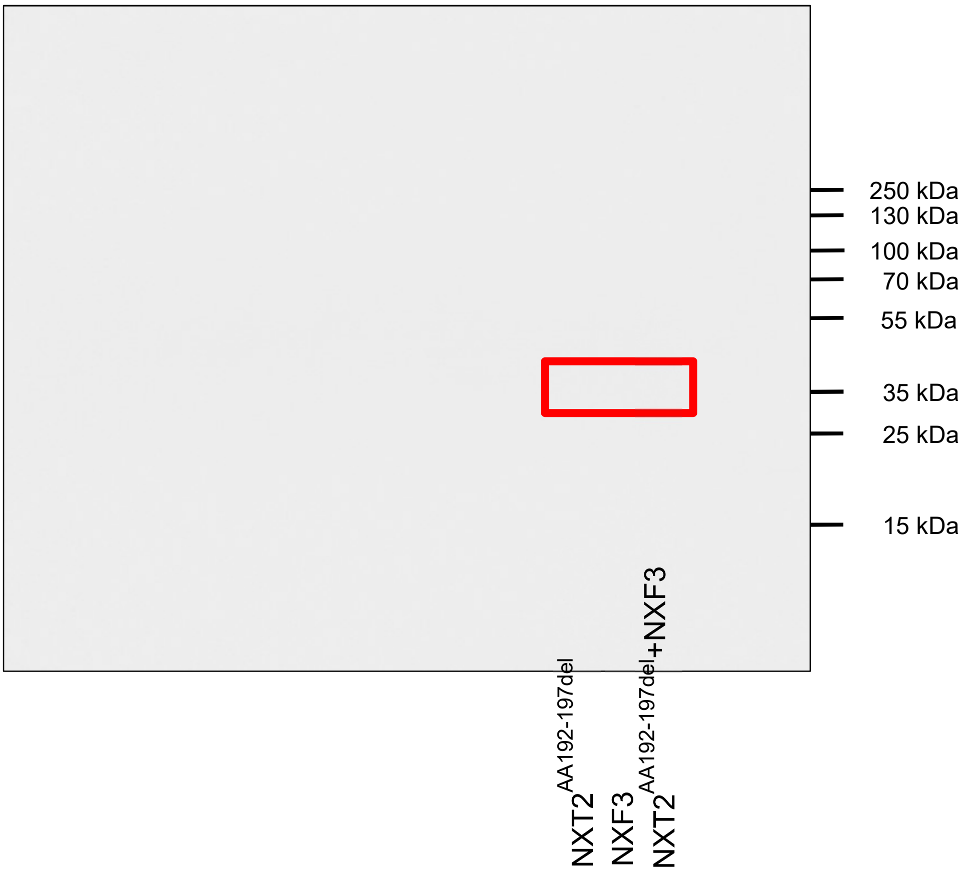

Source Data Supplementary Figure 7a

Lysates

αHA:

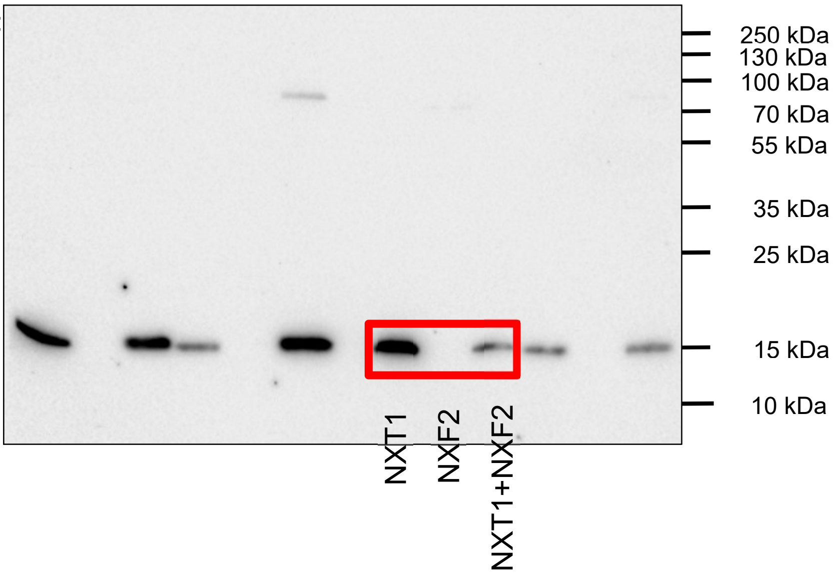

αFLAG:

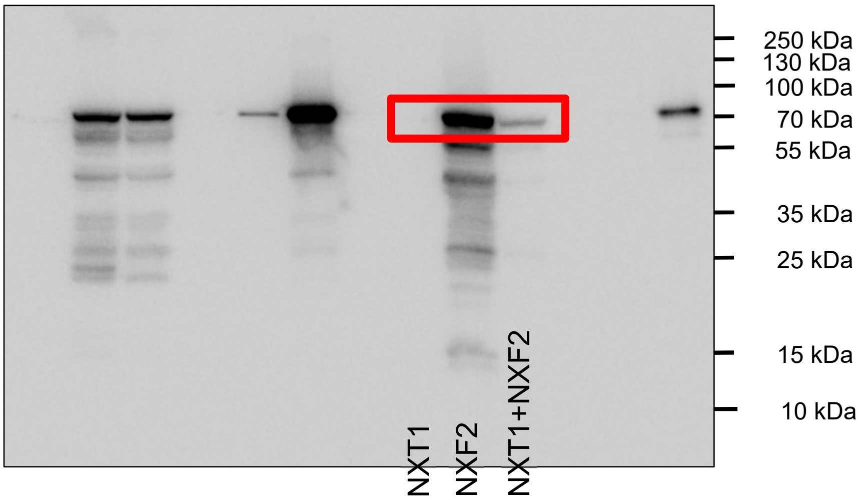

αGAPDH:

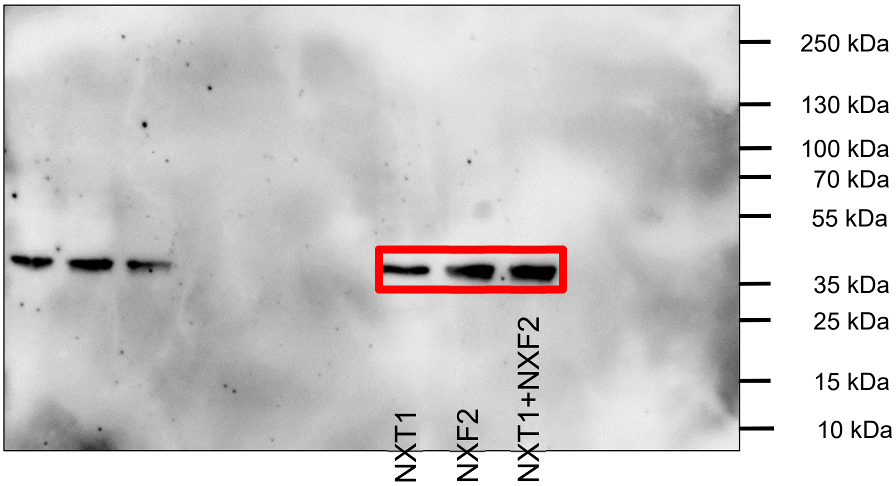

Co-IP

αHA:

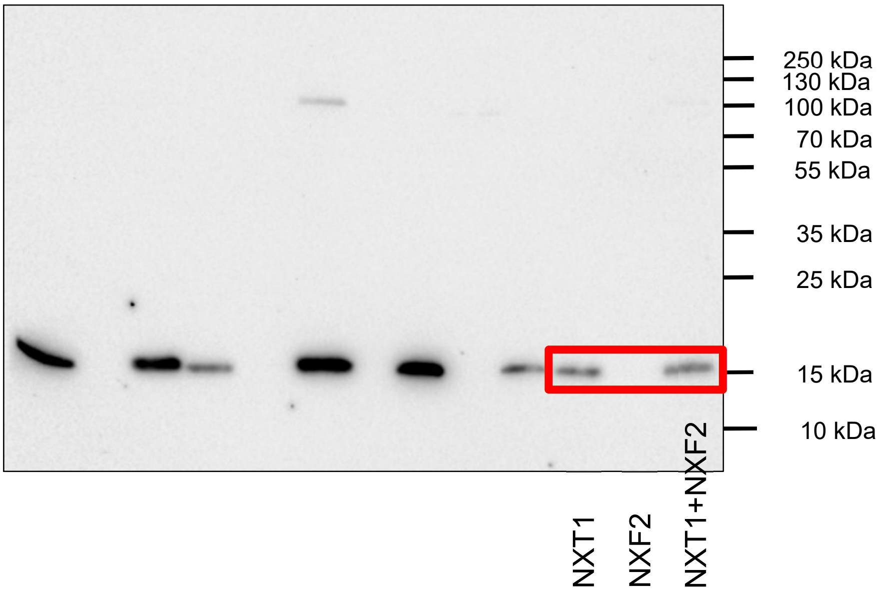

αFLAG:

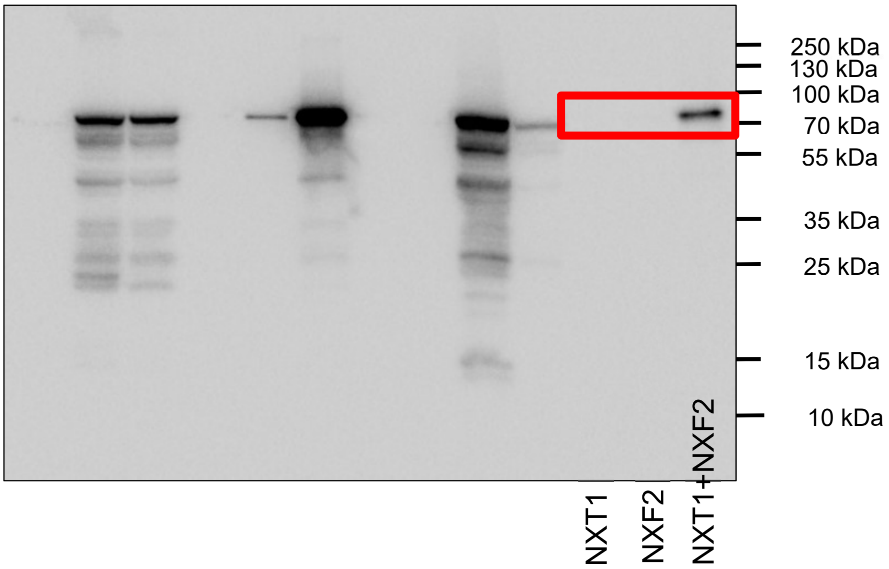

αGAPDH:

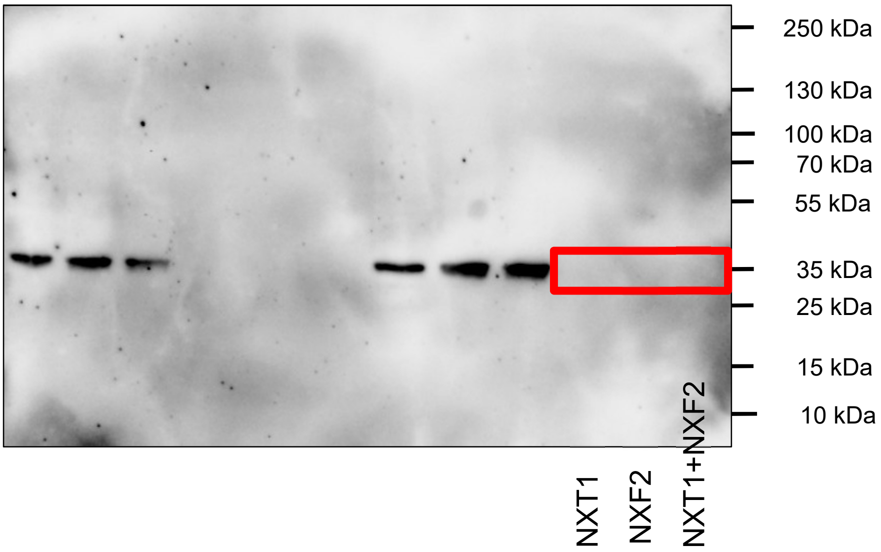

Source Data Supplementary Figure 7b

Lysates

αHA:

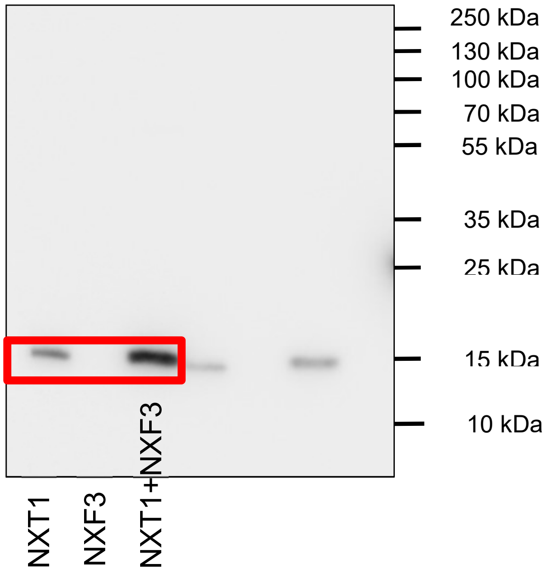

αFLAG:

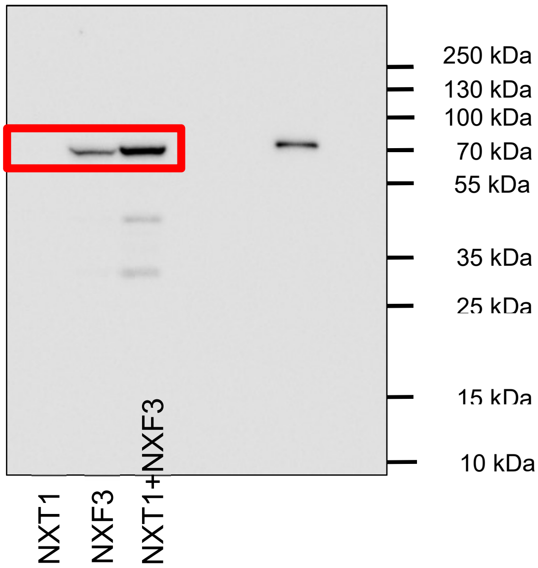

αGAPDH:

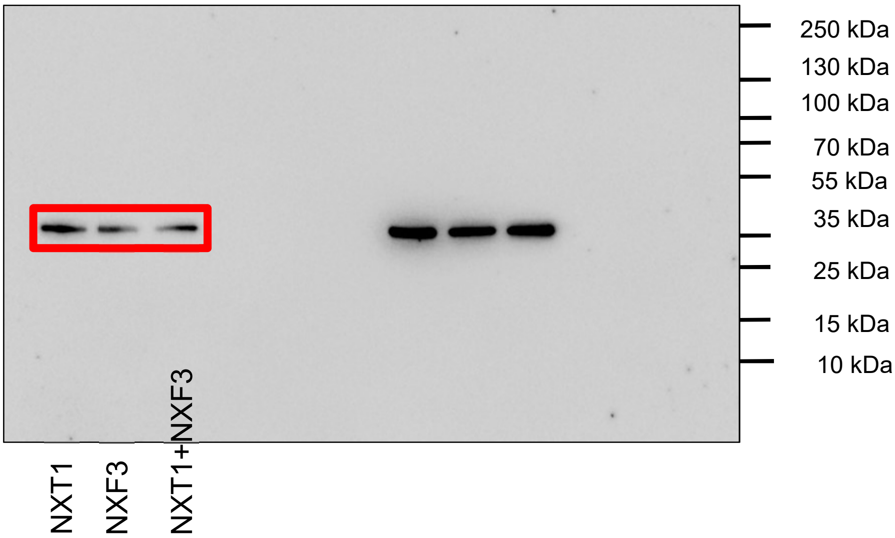

Co-IP

αHA:

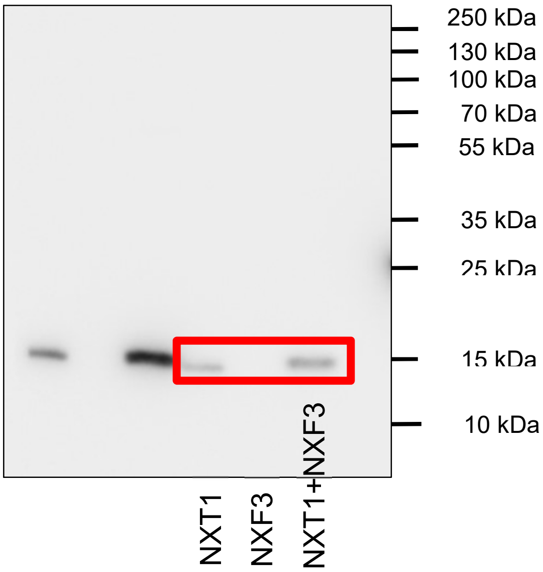

αFLAG:

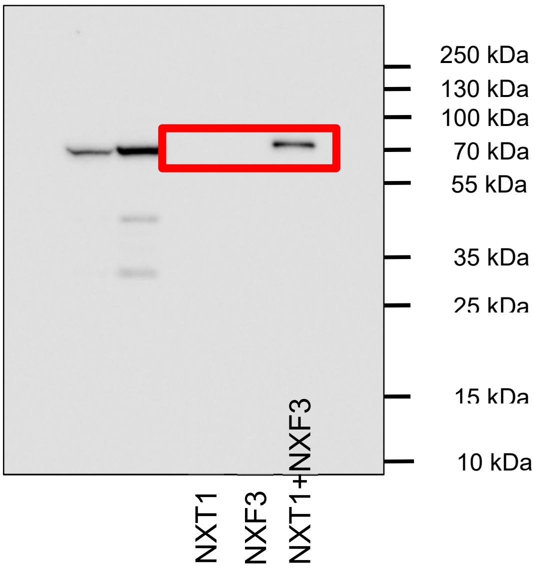

αGAPDH:

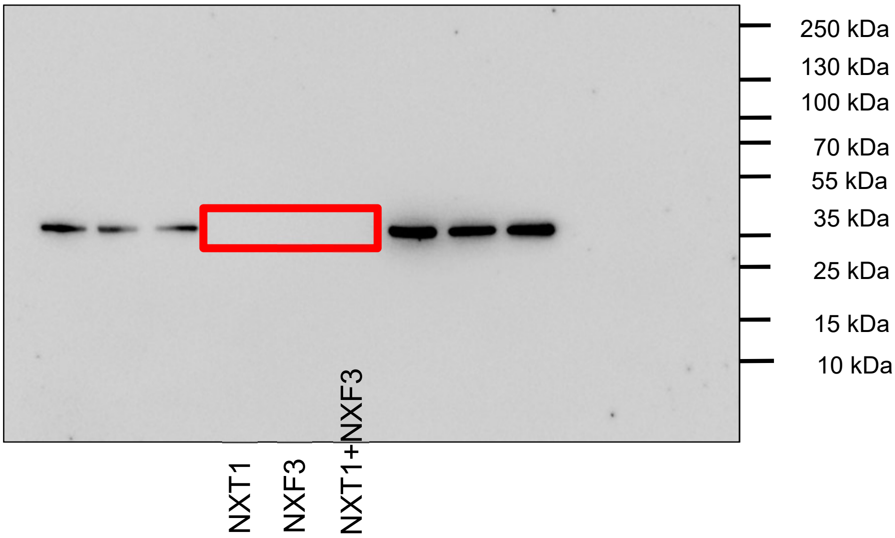

Source Data Supplementary Figure 9e

Lysates

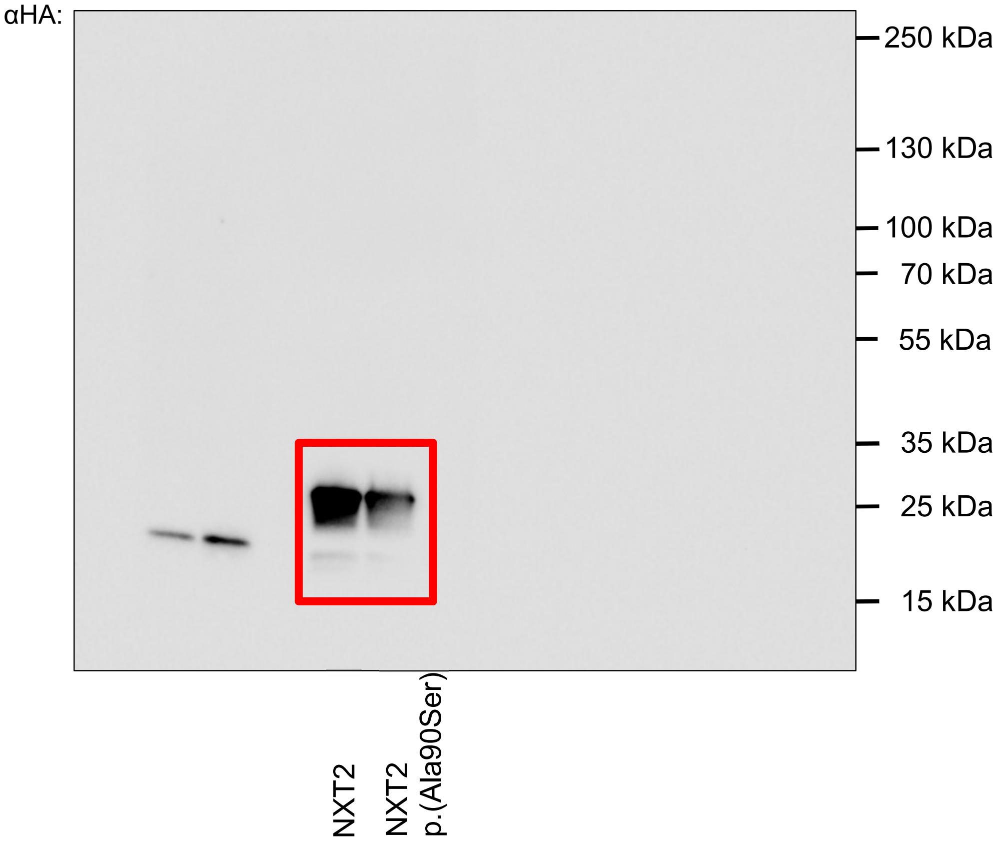

Source Data Supplementary Figure 9f

Lysates

αHA:

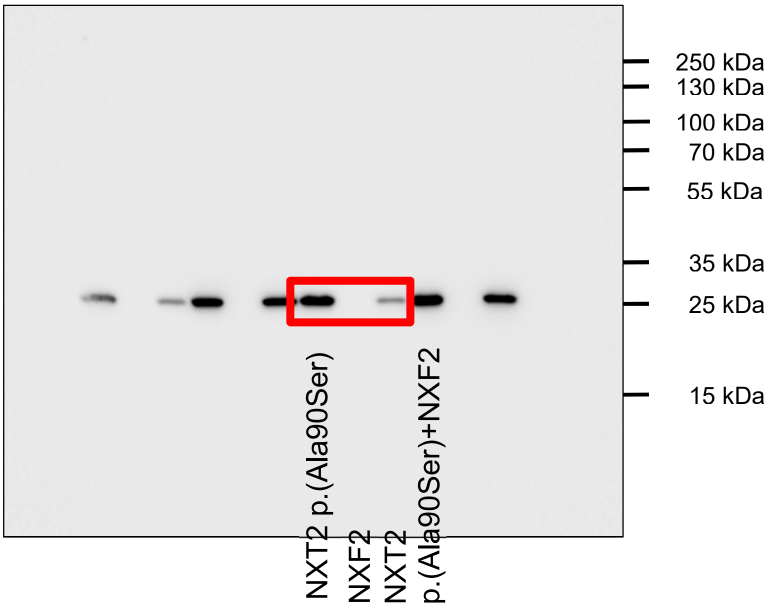

αFLAG:

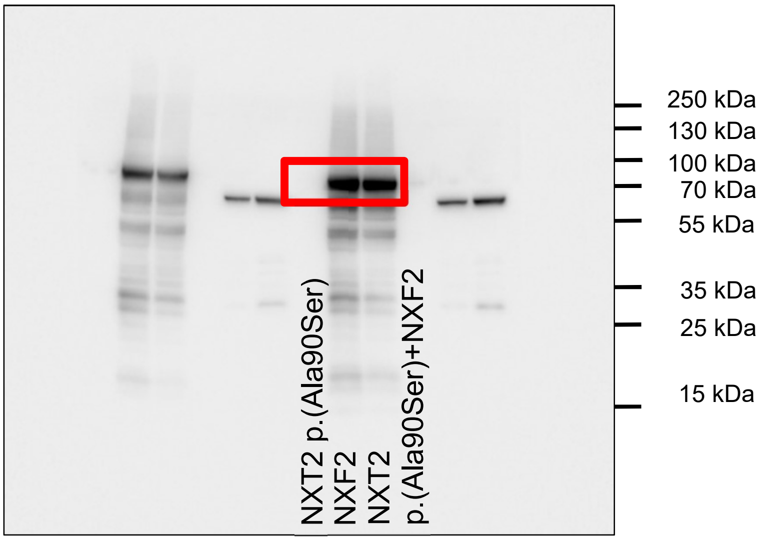

αGAPDH:

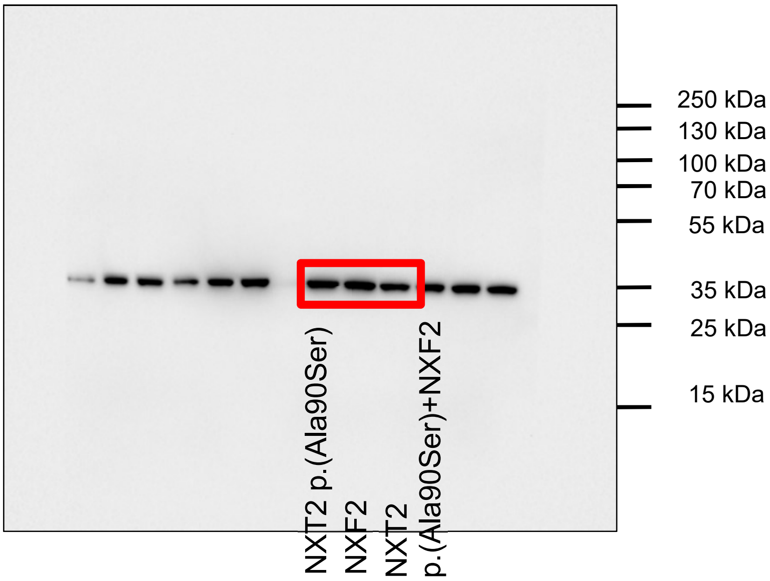

Co-IP

αHA:

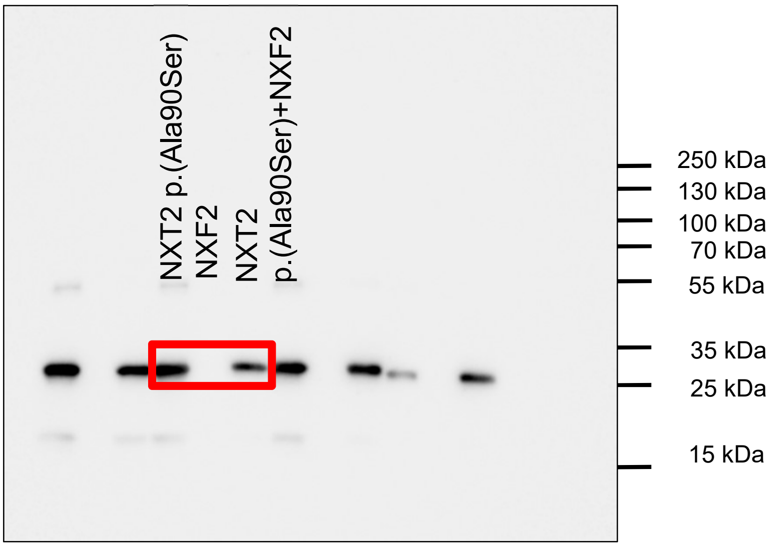

αFLAG:

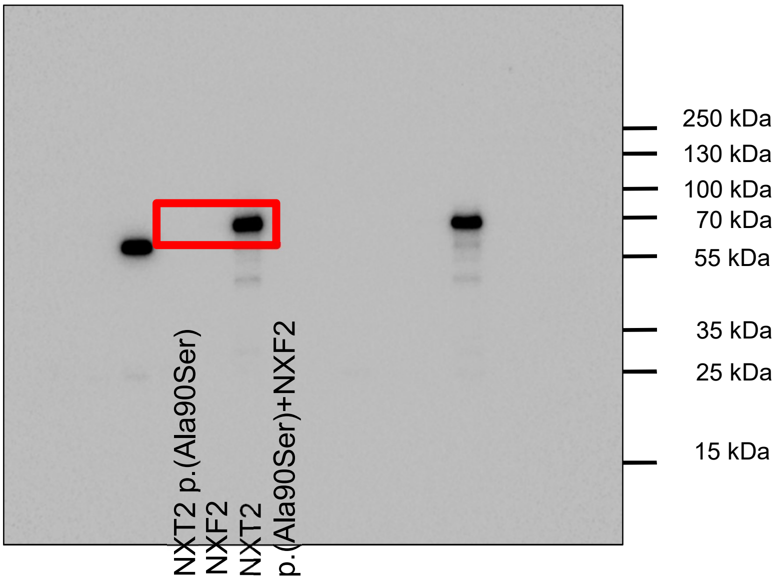

αGAPDH:

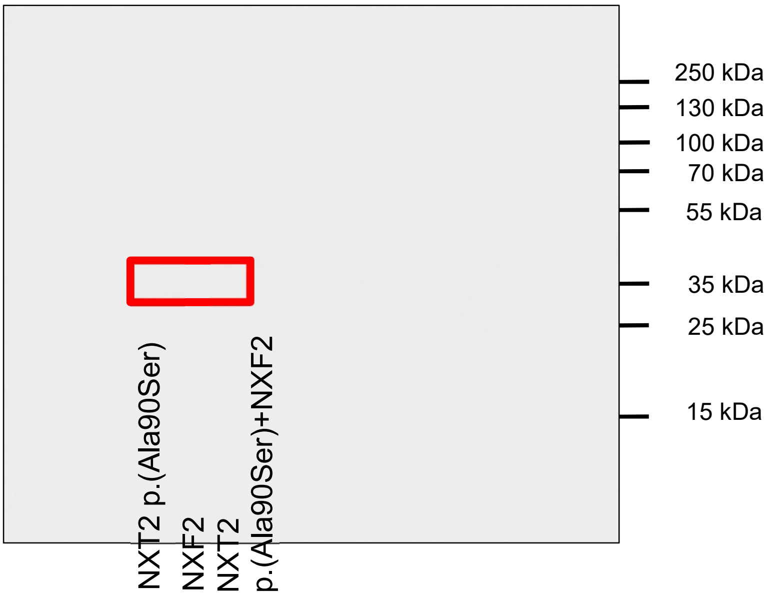

Source Data Supplementary Figure 9g

Lysates

αHA:

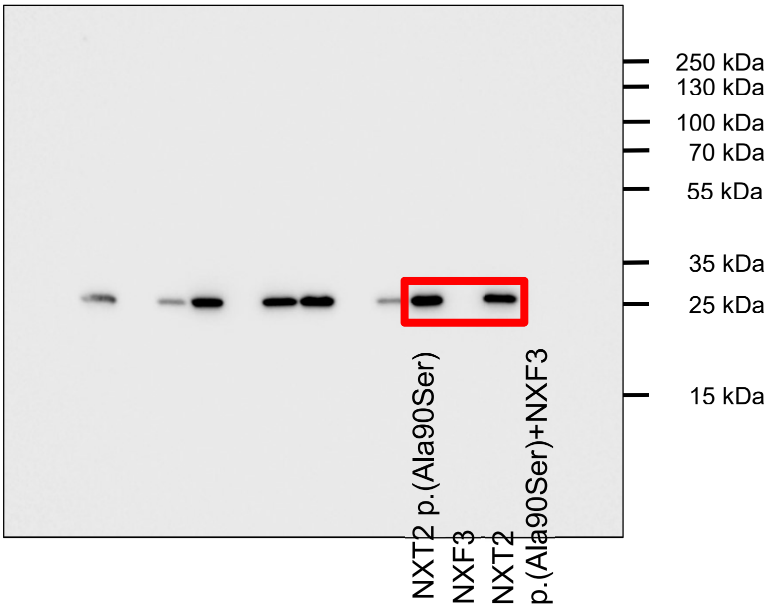

αFLAG:

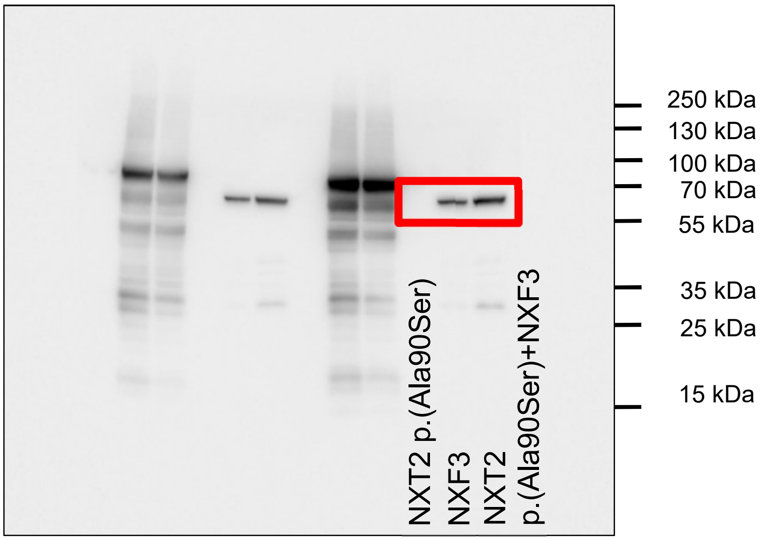

αGAPDH:

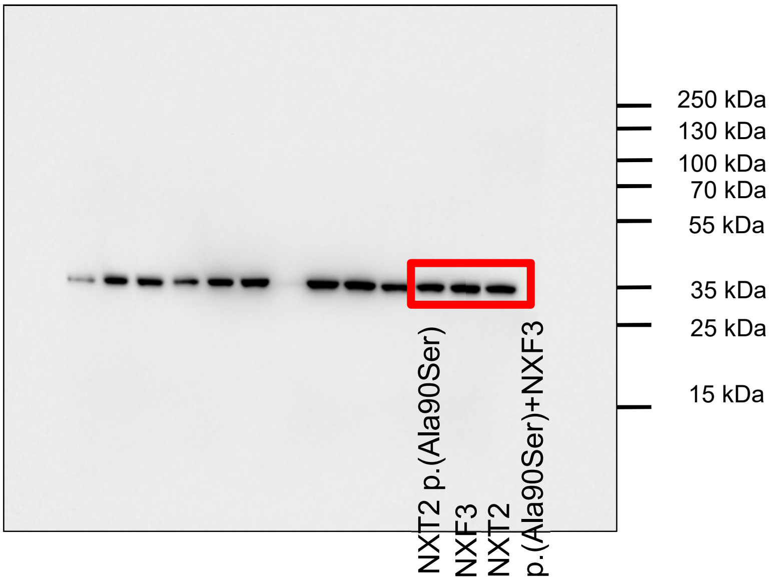

Co-IP

αHA:

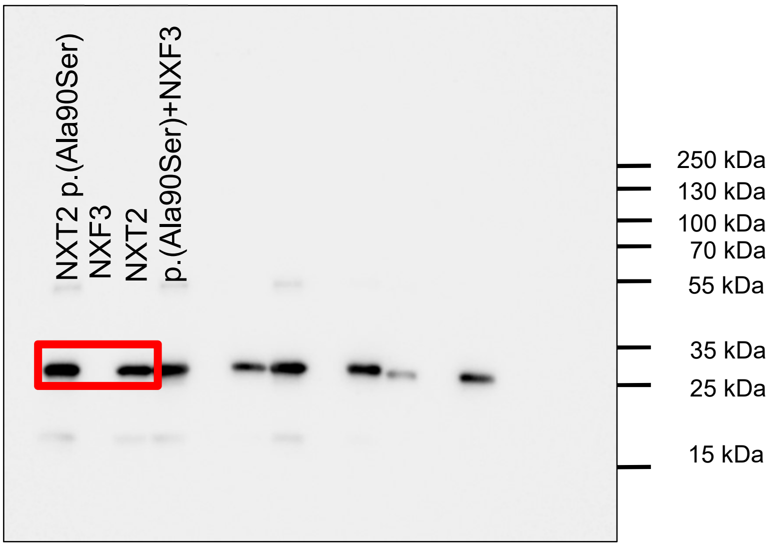

αFLAG:

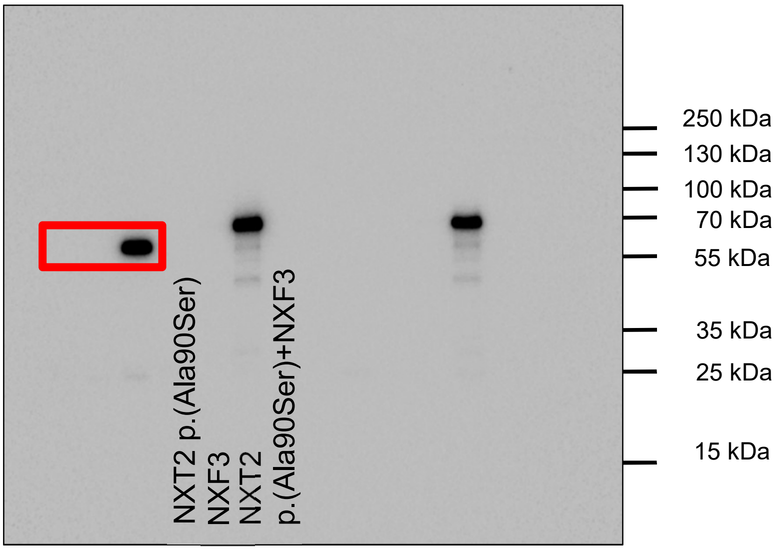

αGAPDH:

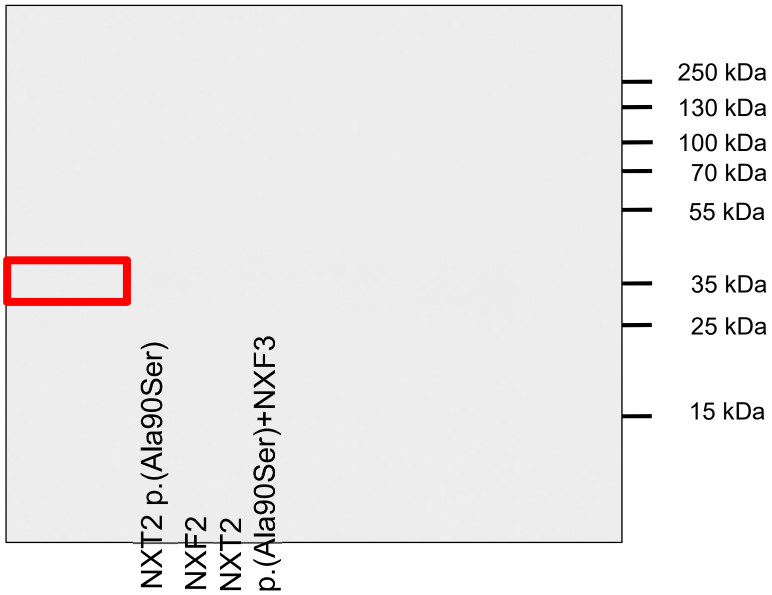

Supplement: Supplementary file 1 — Supplementary Information [file 41467_2025_61463_MOESM1_ESM.pdf]
