# Supplementary material for: NXT2 is a key component of the RNA nuclear export factor complex in the human testis and essential for spermatogenesis
Source: Nat Commun. 2025 Jul 7;16:6254. doi: 10.1038/s41467-025-61463-0 (PMC12234887; doi:10.1038/s41467-025-61463-0)
Supplement: Supplementary file 2 — Description of Additional Supplementary File [file 41467_2025_61463_MOESM2_ESM.pdf]

Description of Additional supplementary files

Supplementary Data 1 (.xls): Abundance values of proteins identified in NXT2 pulldown of testis lysate 1.

Supplementary Data 2 (.xls): At least 2-fold enriched proteins in NXT2 pulldown of testis lysate 1.

Supplementary Data 3: Abundance values of proteins identified in NXT2 pulldown of testis lysate 2.

Supplementary Data 4 (.xls): At least 2-fold enriched proteins in NXT2 pulldown of testis lysate 2.

Supplementary Data 5 (.xls): At least 2-times enriched proteins in NXT2 pulldown of testis lysate 3

Supplementary Data 6 (.xls): Abundance values of proteins detected in NXF3 pulldown of testis lysate 1.

Supplementary Data 7 (.xls): At least 2-fold enriched proteins in NXF3 pull down of testis lysate 1.

Supplementary Data 8 (.xls): At least 2-fold enriched proteins in NXF3 pulldown of testis lysate 2.

Supplementary Data 9 (.xls): Abundance values of proteins detected in NXT1 pulldown of testis lysate 1.
