# Supplementary material for: NXT2 is a key component of the RNA nuclear export factor complex in the human testis and essential for spermatogenesis
Source: Nat Commun. 2025 Jul 7;16:6254. doi: 10.1038/s41467-025-61463-0 (PMC12234887; doi:10.1038/s41467-025-61463-0)
Supplement: Supplementary file 12 — Reporting summary [file 41467_2025_61463_MOESM12_ESM.pdf]

nature portfolio

Corresponding author(s): Birgit Stallmeyer

Last updated by author(s): June 16, 2025

Reporting Summary

Nature Portfolio wishes to improve the reproducibility of the work that we publish. This form provides structure for consistency and transparency in reporting. For further information on Nature Portfolio policies, see our Editorial Policies and the Editorial Policy Checklist.

Statistics

For all statistical analyses, confirm that the following items are present in the figure legend, table legend, main text, or Methods section.

n/a	Confirmed	
☐	☒	The exact sample size (*n*) for each experimental group/condition, given as a discrete number and unit of measurement
☐	☒	A statement on whether measurements were taken from distinct samples or whether the same sample was measured repeatedly
☐	☒	The statistical test(s) used AND whether they are one- or two-sided<br>*Only common tests should be described solely by name; describe more complex techniques in the Methods section.*
☒	☐	A description of all covariates tested
☐	☒	A description of any assumptions or corrections, such as tests of normality and adjustment for multiple comparisons
☒	☐	A full description of the statistical parameters including central tendency (e.g. means) or other basic estimates (e.g. regression coefficient) AND variation (e.g. standard deviation) or associated estimates of uncertainty (e.g. confidence intervals)
☐	☒	For null hypothesis testing, the test statistic (e.g. *F*, *t*, *r*) with confidence intervals, effect sizes, degrees of freedom and *P* value noted<br>*Give P values as exact values whenever suitable.*
☒	☐	For Bayesian analysis, information on the choice of priors and Markov chain Monte Carlo settings
☒	☐	For hierarchical and complex designs, identification of the appropriate level for tests and full reporting of outcomes
☒	☐	Estimates of effect sizes (e.g. Cohen's *d*, Pearson's *r*), indicating how they were calculated

Our web collection on statistics for biologists contains articles on many of the points above.

Software and code

Policy information about availability of computer code

Data collection	no specific software was used for data collection
Data analysis	WES/WGS data analysis: trimming of remaining adapter sequences: Cutadapt v1.1553;Alignment: BWA Mem v0.7.1754 or llumina Dragen Bio-IT platform v4.2. Variant calling: GATK toolkit v3.8 55 or or llumina Dragen Bio-IT platform v4.2. Variant annotation: Ensembl Variant Effect Predictor 56. Mass specrometry analysis: Thermo Xcalibur 4.1 software, Thermo Scientific, Proteome Discoverer 3.1 software (Thermo Fisher Scientific).

For manuscripts utilizing custom algorithms or software that are central to the research but not yet described in published literature, software must be made available to editors and reviewers. We strongly encourage code deposition in a community repository (e.g. GitHub). See the Nature Portfolio guidelines for submitting code & software for further information.

Data

Policy information about availability of data

All manuscripts must include a data availability statement. This statement should provide the following information, where applicable:

- Accession codes, unique identifiers, or web links for publicly available datasets
- A description of any restrictions on data availability
- For clinical datasets or third party data, please ensure that the statement adheres to our policy

Submission of human exome/genome sequencing data from the MERGE cohort to public databases is not covered by the probands informed consent. These data

will be available upon request for academic use and within the limitations of the proband's informed consent from Frank Tüttelmann, Director of the Institute of Reproductive Genetics, Münster, Germany, by contacting frank.tuettelmann@ukmuenster.de. Each request will be reviewed within one month and the researcher will need to sign a data access agreement. Sequencing data from the Nijmegen cohort have been deposited in the European Genome-phenome Archive (EGA) under the accession code EGAS00001005417 [https://ega-archive.org/studies/EGAS00001005417]. These data will be available upon request for academic use and within the limitations of the provided informed consent by applying for access through the EGA's online form. Every request will be reviewed by the Newcastle University Male Infertility Genomics Data Access Committee and the researcher will need to sign a data access agreement after approval. AlphaFold2 structure accession code for NXT2 is AF-Q9NPJ8-F1 [https://alphafold.ebi.ac.uk/entry/Q9NPJ8]. The mass spectrometry proteomics data have been deposited the ProteomeXchange Consortium via the PRIDE63 partner repository with the dataset identifier PXD052904 [ProteomeXchange Dataset PXD052904] and PXD061059 [ProteomeXchange Dataset PXD061059]. Novel genetic variants identified in NXT2 and NXF3 have been deposited in ClinVar with accession numbers SCV005043065 [https://www.ncbi.nlm.nih.gov/clinvar/variation/3235105] , SCV005043066 [https://www.ncbi.nlm.nih.gov/clinvar/variation/3235106], SCV005043067 [https://www.ncbi.nlm.nih.gov/clinvar/variation/3235107] and VCV003602633.1 [https://www.ncbi.nlm.nih.gov/clinvar/variation/3602633]. Source data are provided with this paper.

Research involving human participants, their data, or biological material

Policy information about studies with human participants or human data. See also policy information about sex, gender (identity/presentation), and sexual orientation and race, ethnicity and racism.

Reporting on sex and gender	findings presented in this manuscript apply only to male sex
Reporting on race, ethnicity, or other socially relevant groupings	no analysis was made on the study cohort in respect to race
Population characteristics	most of the patients included in this study are of European origin
Recruitment	All study cohorts are based on a prospective recruitment of men who attended andrological examination because of infertility. Most patients (>90%) were recruited at the Centre of Reproductive Medicine and Andrology (CeRA), University Hospital Münster. In addition patients were recruited at the Department of Obstetrics and Gynecology, Radboud University Medical Center, Nijmegen.
Ethics oversight	All persons gave written consent compliant with local requirements. The study protocol was approved by the local ethics committees: MERGE Münster (2010-578-f-S) and Newcastle: (Newcastle:REC ref. 18/NE/0089), Nijmegen: (NL50495.091.14 version 5.0). Use of testicular tissue for pulldown awas approved by the Münster Ethics Committees/Institutional Review Boards (Ref. No. Münster: 2012-555-f-S and 2010-578-f-S). Use of semen samples from normozoospermic donors was performed according to the protocols approved by the Ethics Committee of the Ärztekammer Westfalen-Lippe and the Medical Faculty Münster (4INie, 2021-402-f-S).

Note that full information on the approval of the study protocol must also be provided in the manuscript.

Field-specific reporting

Please select the one below that is the best fit for your research. If you are not sure, read the appropriate sections before making your selection.

☒ Life sciences ☐ Behavioural & social sciences ☐ Ecological, evolutionary & environmental sciences

For a reference copy of the document with all sections, see nature.com/documents/nr-reporting-summary-flat.pdf

Life sciences study design

All studies must disclose on these points even when the disclosure is negative.

Sample size	For Mass spectrometry sample sizes range from N=7 (NXT2 pulldown) to N=4 (NXF3 pulldown) to N=3 (NXT1 pulldown.). Sample size of IgG pulldown ranges from N=5 (IgG rabbit) to N=3 (IgG mouse). Since human testicular tissue was used for pulldown the sample size is appropriate. In addition key findings on protein-protein interactions were validated by Co-IP of recombinant proteins. Sample size of WES data refers to infertile men of the resepective cohort included in genetic analysis. For MERGE most of this cohort are azoospermic (N = 1,622) or have severely reduced sperm counts: N = 487 with cryptozoospermia (sperm only identified after centrifugation of the ejaculate); N = 168 with extreme oligozoospermia (sperm count <2 million); N = 85 with severe oligozoospermic (sperm count <10 million).
Data exclusions	no data were excluded
Replication	Mass spectrometry for NXT2 and NXF3 pulldown was performed for 2 biological replicates. The replicates confirmed the princple findings of the initial experiment. Mass spectrometry for NXT1 pulldown was performed on 1 biological sample (3 technical replicates). Due to limited access to human testicular tissue and independent replication on a second biological sample was not performed. Main interactions observed in the Mass spectrometry were confirmed b Co-IP of recombinant proteins. All single nucleotide variants described in this manuscript were identified by NGS and confirmed by Sanger sequencing. CNV identified by exome sequencing were confirmed by whole genome sequencing. IHC results that differed from the staining pattern repeatedly seen in testicular tissue with full spermatogenesis were confirmed in at least one additional section. All Western blot experiments were repeated at least three times (N = 3).
Randomization	not applicable

Blinding	For all experiments, the investigators were not blinded to group allocation during data collection and/or analysis but assessment of clinical data and genetic analysis was performed by independent researchers. Mass spectrometry data generation and analysis was also performed by independent researchers from different institutions.

Reporting for specific materials, systems and methods

We require information from authors about some types of materials, experimental systems and methods used in many studies. Here, indicate whether each material, system or method listed is relevant to your study. If you are not sure if a list item applies to your research, read the appropriate section before selecting a response.

Materials & experimental systems

n/a	Involved in the study
☐	☒ Antibodies
☐	☒ Eukaryotic cell lines
☒	☐ Palaeontology and archaeology
☒	☐ Animals and other organisms
☒	☐ Clinical data
☒	☐ Dual use research of concern
☒	☐ Plants

Methods

n/a	Involved in the study
☒	☐ ChIP-seq
☒	☐ Flow cytometry
☒	☐ MRI-based neuroimaging

Antibodies

Antibodies used	α/β-Tubulin, Cell Signaling Technology, #2148, rabbit, polyclonal, LOT: 8<br>FLAG, Merck, #F3165, mouse, monoclonal, LOT: SLCJ3741<br>GAPDH, Cell Signaling Technology, #5174, rabbit, monoclonal, LOT: 8<br>HA, Roche, #11867423, rat, monoclonal, LOT: 65506600<br>MAGEA4, Abcam, #ab139297, mouse, monoclonal, LOT: GR3177245-18<br>NXF3, Merck, #HPA046757, rabbit, polyclonal, LOT: R43903<br>NXT2, Merck, #HPA072010, rabbit, polyclonal, LOT: R102934<br>NXT1, Proteintech, #67680, mouse, monoclonal, LOT: 10017134<br>SMA, Sigma-Aldrich, # A2547 , mouse, monoclonal, LOT: 0000106190<br>DDX4, Abcam, #ab13840, rabbit, polyclonal, LOT: GR3274948-1<br>SOX9, Sigma Aldrich, #AB5535, rabbit, polyclonal, LOT: 3481414
Validation	α/β-Tubulin: https://www.cellsignal.com/products/primary-antibodies/a-b-tubulin-antibody/2148?Ns; application: WB, IP, IHC, IF, ICC; PMID: 37185259; PMID: 37179350<br>FLAG: https://www.sigmaaldrich.com/DE/de/product/sigma/f3165; application: WB, IP, IF, ICC; PMID: 31417089; PMID: 31427575<br>GAPDH: https://www.cellsignal.com/products/primary-antibodies/gapdh-d16h11-xp-rabbit-mab/5174; application: WB, IP, IHC, IF, 6530 citations<br>HA: https://www.sigmaaldrich.com/DE/de/product/roche/roahaha; application: WB, IP, ICC; PMID: 19549692; PMID: 24816145;<br>MAGEA4: https://www.abcam.com/en-de/products/primary-antibodies/magea4-antibody-oti1f9-ab139297#, application: WB, IHC; staining in human testis corresponds to scRNAseq data; PMID: 29555572; PMID: 32382343<br>NXF3: https://www.sigmaaldrich.id/DE/de/product/sigma/hpa046757; application: IF, WB, pulldown; validation: WB (this study, supplementary Figure 1b; IF: staining is absent in sperm of patient with hemizygous LoF variant (this study, Figure 6b)<br>NXT2: https://www.sigmaaldrich.id/DE/de/product/sigma/hpa072010; application: IHC, WB, pulldown; validation: WB (this study, Figure S1a and IHC: staining pattern corresponds to scRNAseq data; staining is absent in testicular tissue of patient with hemizygous LoF variant (this study, Figure 4d).<br>NXT1: https://www.ptglab.com/products/NXT1-Antibody-67680-1-Ig.htm; application: IHC, WB, pulldown; validation: WB (this study, supplementary Figure 1c)<br>SMA: https://www.sigmaaldrich.com/DE/de/product/sigma/a2547; application: IHC; validation: e.g. PMID: 31208912, PMID22966006;<br>DDX4: https://www.abcam.com/en-us/products/primary-antibodies/ddx4-mvh-antibody-ab13840; application: IHC; validation: e.g. PMID: 39226347, PMID: 38017073.<br>SOX9: https://www.sigmaaldrich.com/DE/de/product/sigma/hpa001758?; application: IHC, IF; staining in human testis corresponds to scRNAseq data; 49 citations, e.g. PMID: 30542406, PMID: 29897331

Eukaryotic cell lines

Policy information about cell lines and Sex and Gender in Research

Cell line source(s)	HEK293T Lenti-X, Clontech Laboratories; catalog number: 632180
Authentication	none of the cells were authenticated
Mycoplasma contamination	cell lines were negative for mycoplasm contamination

Commonly misidentified lines (See ICLAC register)	no commonly misidentified cell lines were used

Plants

Seed stocks	not applicable
Novel plant genotypes	not applicable
Authentication	not applicable
